# Selection and Characterisation of Minor Histocompatibility Antigen‐Specific Regulatory T Cells in Fully HLA‐Matched Setting for GVHD Therapy

**DOI:** 10.1002/eji.70096

**Published:** 2025-12-18

**Authors:** Carolina Paulino Pacini, Rita I. Azevedo, Luís Ramalhete, Hugo A. J. Lainé, Maria V. D. Soares, João F. Lacerda

**Affiliations:** ^1^ JLacerda Lab Gulbenkian Institute for Molecular Medicine Lisbon Portugal; ^2^ Blood and Transplantation Center of Lisbon Instituto Português do Sangue e da Transplantação Lisbon Portugal; ^3^ NOVA Medical School Universidade NOVA De Lisboa Lisbon Portugal; ^4^ inova4health‐Advancing Precision Medicine Núcleo De Investigação em Doenças Renais NOVA Medical School Faculdade De Ciências Médicas Universidade NOVA De Lisboa Lisbon Portugal; ^5^ Advanced Data Analysis Gulbenkian Institute for Molecular Medicine Lisbon Portugal; ^6^ Faculdade De Medicina Universidade De Lisboa Lisbon Portugal; ^7^ Serviço De Hematologia e Transplantação De Medula ULS Santa Maria Lisbon Portugal

**Keywords:** antigen‐specific Treg, cGVHD, HLA class II, HLA‐matched, mHA

## Abstract

Graft‐versus‐host disease is mediated by donor‐derived T cells reactive against the recipient's broadly expressed minor histocompatibility antigens (mHA). Regulatory T cells (Treg) have been explored as a therapeutic approach for chronic GVHD (cGVHD). The promising results from polyclonal Treg trials in this setting have led us to develop a Treg product specific for mismatched minor antigens between patient and donor (mTreg), circumventing broad immune suppression risks. HLA‐matched siblings of opposite sexes were used to obtain the sister's CD4^+^CD25^hi^CD127^low^ Treg for co‐culture with the respective brother's dendritic cells as a source of mismatched mHA. We have established the optimal culture conditions resulting in the highest mTreg proliferation and viability. Comprehensive phenotyping during the ex vivo selection shows PD‐1, CTLA‐4, CD39 and HLA‐DR expression. Transcriptomic analysis revealed a switch in metabolic process, and up‐regulation of functional Treg genes. Furthermore, mTreg possess specific and potent suppressive activity, in which there is a dependency on cell‐to‐cell contact and a role for HLA class II expression on mTreg. This protocol would allow the generation of Treg specific to an array of mHA from the recipient's healthy tissues, likely providing a directed and strong suppression of cGVHD.

## Graphical abstract

We optimised a protocol for mHA‐specific Treg (mTreg) selection in an HLA‐matched context while defining its phenotype, transcriptional state and function. mTreg were highly activated and exerted specific, HLA class II‐, contact‐dependent suppression. This protocol can be explored as a highly personalised antigen‐specific Treg‐based therapy in future clinical trials for cGVHD.

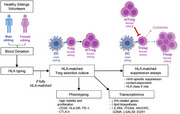

**Abbreviations**
allo‐HSCT: allogeneic hematopoietic stem cell transplantation
CAR: chimeric antigen receptors
cGVHD: chronic graft‐versus‐host disease
DC: dendritic cells
GVHD: graft‐versus‐host disease
HLA: human leukocyte antigen
H‐Y: histocompatibility‐Y chromosome minor antigens
mHA: minor histocompatibility antigens
moDC: monocyte‐derived dendritic cells
mTreg: mHA‐specific regulatory T cells
PBMC: peripheral blood mononuclear cells
Tcon: conventional T cells
Treg: regulatory T cells

## 1 Introduction

Allogeneic hematopoietic stem cell transplantation (allo‐HSCT) is the only curative therapy for many patients with haematological malignancies. Allo‐HSCT consists of the replacement of host haematopoiesis by a healthy hematopoietic system derived, ideally, from an HLA‐matched donor. In haematological malignancies, the success of allo‐HSCT relies on the recognition of residual malignant cells by donor‐derived immune cells, a process called graft‐versus‐leukaemia (GVL) effect [1]. Nonetheless, even in patient–donor pairs matched for the main 10 major HLA alleles, disparities in minor Histocompatibility Antigens (mHA) can lead to reactivity of donor‐derived lymphocytes against tissues of the patient [2], causing graft‐versus‐host disease (GVHD). GVHD is a major cause of morbidity and mortality after allo‐HSCT [3]. The gold‐standard treatment for chronic GVHD (cGVHD) includes prolonged courses of broadly immunosuppressive drugs, which can interfere with GVL and/or lead to infection‐related mortality [4]. Therefore, targeted therapies for GVHD that decrease nonspecific immune suppression are a clear unmet need [5, 6].

Regulatory T cells (Treg) are naturally occurring immunosuppressive T cells constitutively expressing high levels of CD25 and Foxp3 [7]. Tregs have been shown to play an essential role in the establishment of immune tolerance after allo‐HSCT, as demonstrated by the impaired Treg reconstitution in patients with cGVHD [8, 9, 10, 11]. These observations have led to studies aiming to replenish this population to restore immune tolerance control. Different strategies for Treg manipulation have been explored for the prevention or treatment of GVHD [12, 13, 14, 15, 16, 17, 18]. We have coordinated the EC‐funded consortium TREGeneration [18] to investigate the safety and efficacy of fresh donor‐derived Treg infusion in moderate/severe cGVHD in phase I/II clinical trials, in which patients receiving higher doses of Treg showed better clinical responses [18].

Despite the promising results in some of the clinical trials performed over the last decade [13, 15, 16, 17, 18, 19, 20, 21], they relied on the activity of polyclonal Treg, which bear the potential of broad immunosuppression. Alternatively, alloantigen‐specific Treg have shown potential to provide a specialised, overall more efficient GVHD suppression than polyclonal Treg [22, 23, 24]. Still, current studies focus mainly on the expansion of monoclonal Treg as the source for an antigen‐specific Treg product [25, 26, 27], which may not be sufficient for optimal results in a complex disease involving reactivity against several mHA. Importantly, the antigen‐specific Treg generated for clinical use should be investigated for their functional aspects.

Therefore, the selection and expansion of a Treg population targeting several mHA involved in cGVHD will likely provide a more efficient and patient‐oriented cell therapy. Hence, we sought to establish a protocol for obtaining mHA‐specific Treg (mTreg) in a full HLA‐matched setting, using fully HLA‐matched donor pairs (12/12 major HLA), while allowing for disparities in mHA by choosing pairs of opposite sexes. Thus, to replicate the allo‐HSCT HLA‐matched context, we recruited fully HLA‐matched healthy siblings. The chances of a full match of HLA genes between siblings are 25%. Purified CD3^+^CD4^+^CD25^hi^CD127^low/−^ Treg cells from the female sibling were co‐cultured with monocyte‐derived dendritic cells (moDC) from the respective fully HLA‐matched male sibling using low concentrations of IL‐2 in xeno‐ and serum‐free conditions to select mTreg. We evaluated the phenotype and transcriptomic profile of mTreg throughout the co‐culture period. At the end of the co‐culture, we investigated the mTreg suppressive activity and its underlying mechanisms. We found that mTreg preserved their viability and expression of Foxp3 and CD25, upregulated activation and functional genes, and exhibited specific and potent suppressive function that was dependent on cellular contact and involved HLA class II expression on mTreg.

## 2 Results

### 2.1 Optimisation of the mTreg Selection Protocol

Selection of Treg specific against mHA was performed by co‐culturing peripheral blood Treg from a female donor (hereafter referred to as ‘sister’) with moDC from their HLA‐matched male sibling (hereafter referred to as ‘brother’). The sex difference between 100% HLA‐matched donors ensures the presence of distinct Y‐chromosome‐associated mHA in males but not in females. Within the 43 pairs of siblings recruited and HLA‐typed, 14 were fully HLA‐matched. The haplotypes obtained by HLA high‐resolution sequencing are shown in Table S1.

CD14^+^ monocytes were purified from peripheral blood mononuclear cells (PBMC) to generate moDC. These were used as mHA‐mismatched antigen‐presenting cells in the Treg selection culture. Monocyte differentiation into dendritic cells resulted in over 90% conversion to the CD14^−^CD11c^+^ phenotype (Figure S1A). After stimulation with a gold‐standard cytokine cocktail, moDC upregulated the activation and functional markers HLA‐DR, CD80, CD86, PD‐L1 and CD83 (Figure S1B,C). These activated moDC are referred to hereafter as ‘DC’.

**FIGURE 1:**
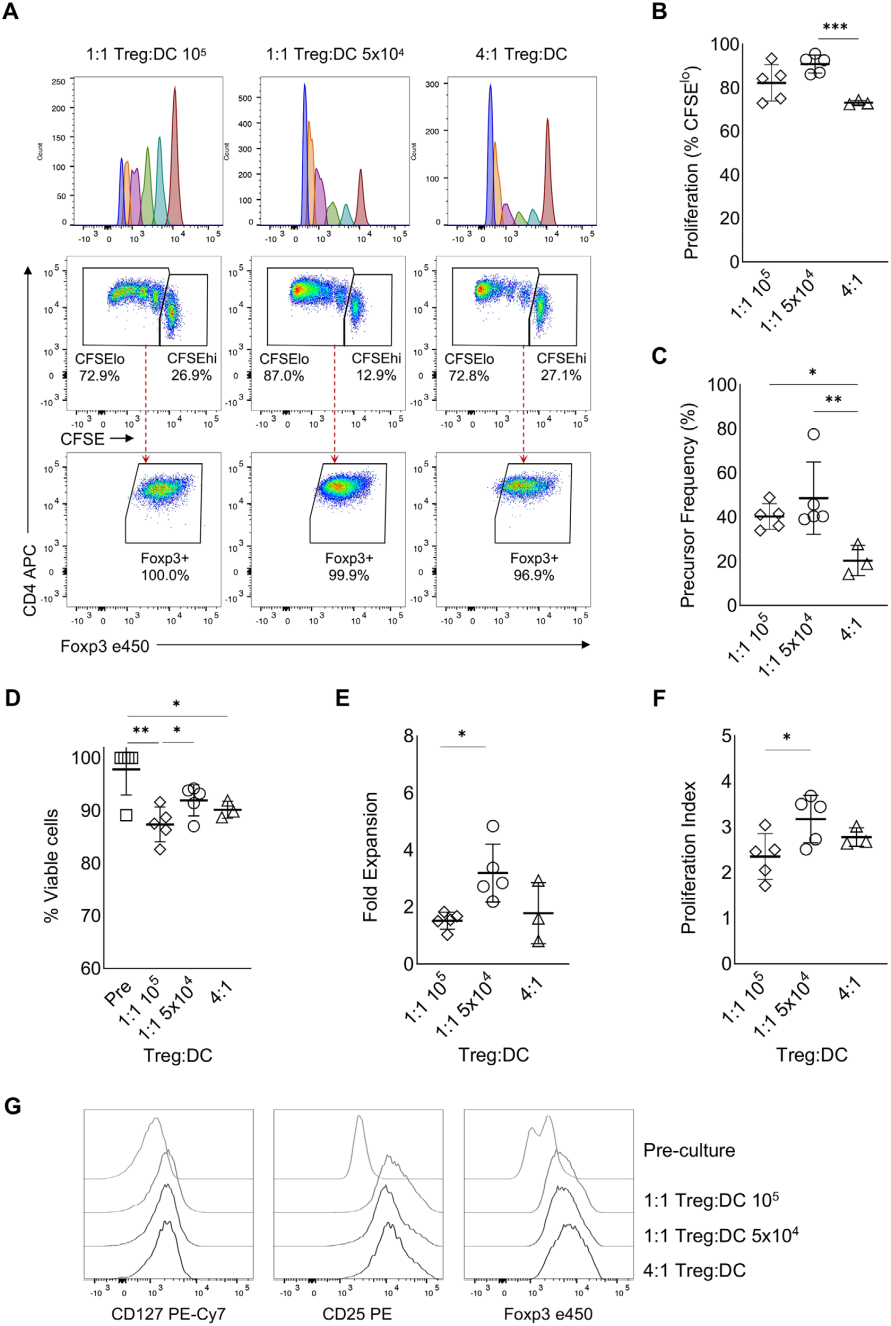
mTreg selection and proliferation in a fully HLA‐matched setting. Female sibling Treg were isolated and co‐cultured with mature DC from her HLA‐matched brother in the presence of IL‐2, IL‐15, and rapamycin for 14 days. Different ratios of Treg and DC were tested and analysed at the end of the culture. (A) Representative pseudo‐colour plots of CFSE dilution by proliferation modelling (top panel) or frequency of CFSE^low^ (middle panel) and Foxp3^+^ cells (bottom panel) after 14 days when 10^5^ Treg were seeded with 10^5^ DC (1:1 Treg:DC 10^5^); 5 × 10^4^ Treg were seeded with 5 × 10^4^ DC (1:1 Treg:DC 5 × 10^4^), or 8 × 10^4^ Treg were seeded with 2 × 10^4^ DC (4:1 Treg:DC). At the end of the culture, each Treg:DC ratio was analysed for the frequency of CFSE^low^ cells within CD3^+^CD4^+^ population (B), precursor frequency (C), frequency of viable cells (viability dye‐negative cells within CD3^+^CD4^+^ population) (D), fold expansion (calculated by dividing the number of mTreg at the end of culture by those seeded on Day 0) (E), and proliferation index (F). (G) Overlay histograms display CD127, CD25 and Foxp3 expression within the undivided CFSE^+^ population on Day 0 (pre‐culture) and within the proliferated CFSE^low^ mTreg population on Day 14 in each cell ratio tested. (B–F) Mean + SD of data obtained from three (4:1 condition) to five (1:1 conditions) independent experiments with a different pair of siblings each are shown. Symbols represent each experiment. The statistical models used were Welch one‐way ANOVA (B), Kruskal–Wallis followed by Dunn's test (C, D) and one‐way ANOVA (E, F). *p < 0.05; **p < 0.01; ***p < 0.001.

Treg from the sister were isolated by FACS as CD3^+^CD4^+^CD25^hi^CD127^low^ cells (Figure S2). After 14 days of in vitro co‐culture with the respective HLA‐matched brother's DC in the presence of IL‐2, IL‐15 and rapamycin, sister‐derived Treg reacting to disparate mHA (mTreg) from the brother proliferated. We compared three different Treg:DC ratios: 1:1 containing 10^5^ Treg + 10^5^ DC; 1:1 with 5 × 10^4^ Treg + 5 × 10^4^ DC; and 4:1 with 8 × 10^4^ Treg + 2 × 10^4^ DC. The 4:1 condition resulted in the lowest Treg proliferation as assessed by CFSE dilution (mean 73.2% of CD3^+^CD4^+^CFSE^low^ versus 90.9% in 1:1 5 × 10^4^ condition versus 82.3% in 1:1 10^5^ condition) (Figure 1A,B). We further calculated precursor frequency (PF), which defines the fraction of the original Treg that responded and divided at least once during the 14‐day culture period. PF was lowest at 4:1 Treg:DC ratio (mean 20.3% versus 48.6% in 1:1 5 × 10^4^ condition versus 40.3% in 1:1 10^5^ condition) (Figure 1C). This result indicates that the 4:1 Treg:DC ratio may not be ideal due to the scarcity of mHA. On the other hand, the 1:1 ratio containing 10^5^ Treg + 10^5^ DC presented the lowest CD4 T cell viability, likely caused by some level of competition for space and nutrients (Figure 1D).

Within the 1:1 Treg:DC ratio, the condition containing 5 × 10^4^ of each population resulted in the highest fold expansion (Figure 1E) and proliferation of responding Treg as measured by the proliferation index (PI, the average number of divisions that all responding cells have undergone throughout the culture period; mean 3.17 in 1:1 5 × 10^4^ condition versus 2.35 in 1:1 10^5^ condition) (Figure 1F). In all conditions, CD127 expression remained unaltered, Foxp3 was highly expressed and CD25 was upregulated in the CFSE^low^ proliferated fraction due to mTreg activation (Figure 1G).

Therefore, considering the superior outcomes in proliferation, fold expansion and viability, we established the 1:1 ratio with 5 × 10^4^ of Treg and DC as the optimal dose for selecting mTreg. Moreover, we compared two different IL‐2 concentrations, 10 U/mL and 100 U/mL, which were equally efficient in inducing mTreg proliferation (data not shown). Thus, we adopted the 5 × 10^4^ Treg + 5 × 10^4^ DC 1:1 ratio and 10 U/mL IL‐2 as the standard for the selection of mTreg.

### 2.2 mTreg Show an Activated Phenotype and Stable Foxp3 Expression

We examined the expression of classical Treg markers during mTreg selection culture by phenotyping fresh Treg on Day 0 (pre‐selection) and at Days 3, 7 and 14 of the co‐culture with fully HLA‐matched DC. For this analysis, cells were not labelled with CFSE, which can alter expression patterns ([28] and unpublished observations). As expected, Treg remained CD127^low^, in contrast to Tcon cultured in parallel with the same HLA‐matched DC but without rapamycin. CD25 was up‐regulated early and sustained high expression levels on mTreg until Day 14, in contrast to Tcon (Figure S3A). Although Tcon transiently expressed Foxp3 from Days 3 to 7 of HLA‐matched activation, Foxp3 remained highly expressed in mTreg at all time points (Figure S3A,B).

**FIGURE 2:**
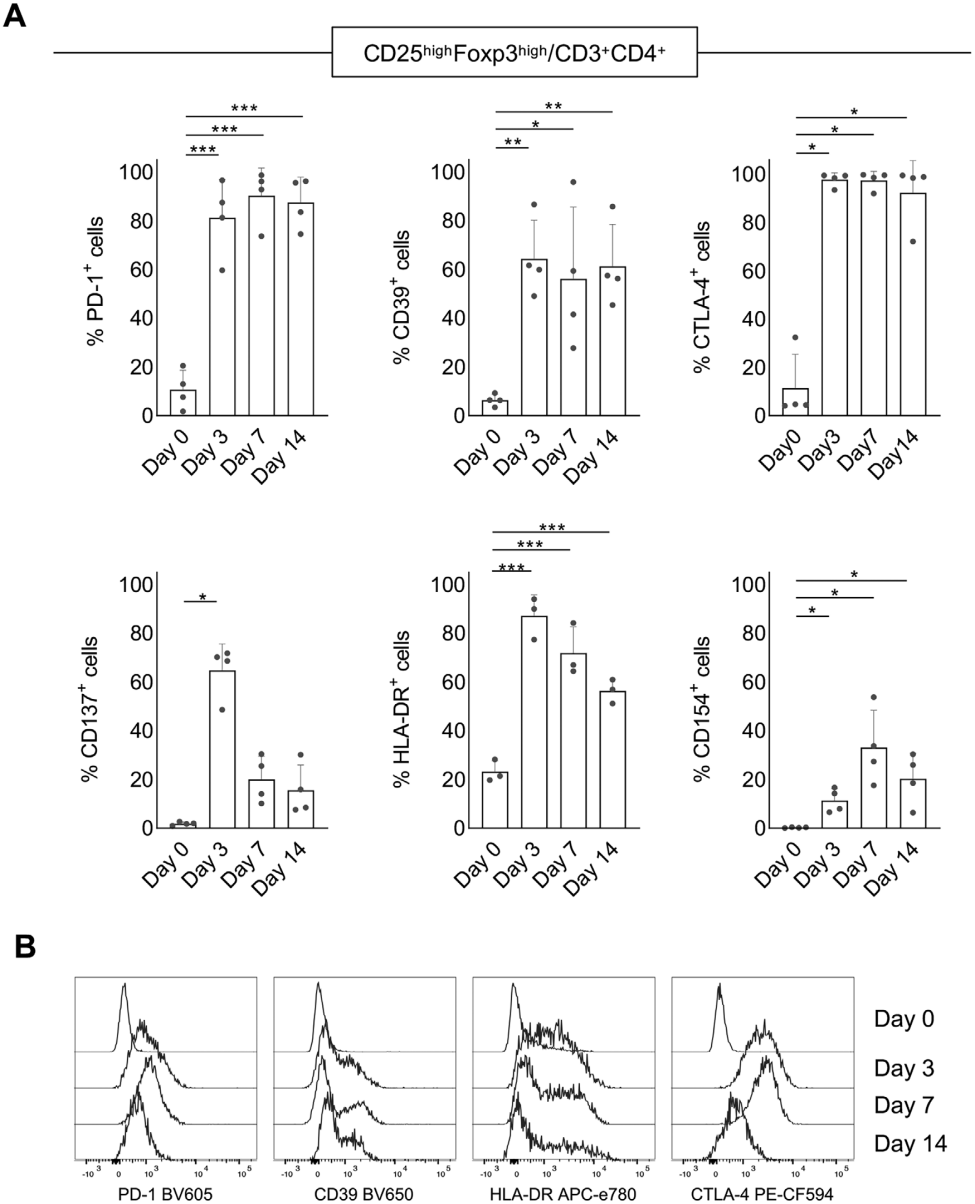
CD25^hi^Foxp3^hi^ mTreg activation and functional marker expression during fully HLA‐matched selection culture. Female sibling Treg were co‐cultured with male sibling HLA‐matched DC at 1:1 Treg:DC 5 × 10^4^ ratio as described. Phenotyping of CD3^+^CD4^+^CD25^hi^Foxp3^hi^ Treg cells was performed by flow cytometry on Day 0 (pre‐selection culture), Days 3, 7 and 14 of the selection culture. (A) Frequency of PD‐1^+^, CD39^+^, CTLA‐4^+^, CD137^+^, HLA‐DR^+^ and CD154^+^ cells within the CD3^+^CD4^+^CD25^hi^Foxp3^hi^ population. Mean + SD of data (bars) obtained from three to four independent experiments using different pairs of siblings in each are shown. Symbols represent each experiment. The statistical models used were Welch one‐way ANOVA (for CD39, CD154), Kruskal–Wallis followed by Dunn's test (for CD137, CTLA‐4) and one‐way ANOVA (for PD‐1, HLA‐DR) *p < 0.05; **p < 0.01; ***p < 0.001. (B) Representative histogram overlays of PD‐1, CD39, HLA‐DR and CTLA‐4 expression within the CD3^+^CD4^+^CD25^hi^Foxp3^hi^ population per timepoint.

**FIGURE 3:**
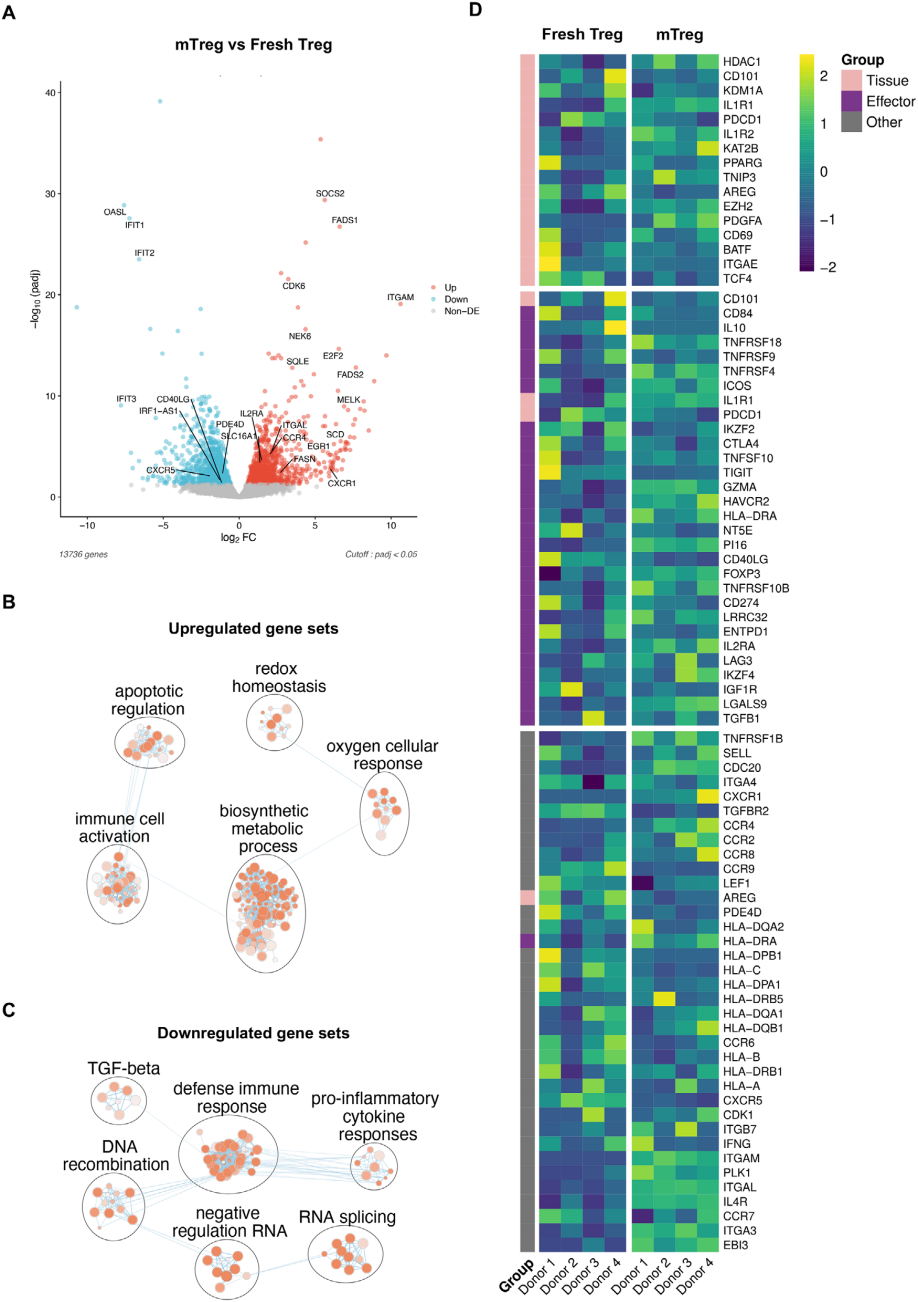
mTreg upregulated activation, functional and metabolic‐associated genes after selection culture with HLA‐matched stimuli. Differential gene expression analysis was performed comparing fresh Treg cells pre‐culture (Day 0) and after the selection culture (Day 14), as mTreg, from four different female siblings. (A) Volcano plot of the differentially expressed genes on mTreg compared to fresh Treg (padj < 0.05, log2FC > 0.585). Red represents up‐regulation; blue represents down‐regulation; and Grey represents non‐significant change in expression. (B, C) Enrichment maps generated by Cytoscape (v3.10.3) and Enrichment Map (v3.5.0) from g:Profiler data on Gene Ontology terms (biological process). Nodes are gene sets which are circled and labelled according to their similarity based on their related genes. The size of the node is proportional to the total number of genes within each gene set. Proportion of shared genes between pathways represented as the thickness of the edges between nodes. Red colour gradient of the node is related to the level of enrichment significance (FDR *q* < 0.05). Simplified network after manually filtering out general and uninformative sub‐networks is displayed within mTreg upregulated (B) and downregulated (C) genes. (D) Heat‐map of gene expression related to tissue Treg genes, effector Treg genes or other relevant genes in fresh Treg (Day 0) or mTreg (Day 14) group.

Treg activation induced the up‐regulation of PD‐1, CD39, CTLA‐4 and HLA‐DR at Day 3. These markers remained high until the end of the culture on CD3^+^CD4^+^CD25^hi^Foxp3^hi^ mTreg cells (Figure 2A). Their Median Fluorescence Intensity (MFI) was also increased or maintained when compared to the fresh isolated Treg on Day 0 (Figure S4). Of note, HLA‐DR expression level stayed high within HLA‐DR^+^ mTreg cells (Figure 2B). CD137 is an early activation marker that was transiently expressed, peaking at Day 3 (Figure 2A), but decreasing in expression afterwards. On the other hand, CD154 expression remained low at all time points examined, both in frequency (Figure 3A) and in MFI (Figure S4). Considering the whole CD3^+^CD4^+^ population, PD‐1, CD39, CTLA‐4 and HLA‐DR were still upregulated (Figure S5A). There was no significant statistical difference in CD154 expression when compared to Day 0, and the majority of CD3^+^CD4^+^ cells expressed CTLA‐4 at the end of the culture (Figure S5B). Importantly, on Day 14, mTreg expressed higher levels of PD‐1 and CTLA‐4 than Tcon from the same donor co‐cultured in parallel with the same HLA‐matched DC, but without rapamycin (Figure S5C).

We analysed the co‐culture supernatants at different time points to quantify cytokines that have been described to play a role in Treg function and in mediating immune responses. We observed that the levels of TGF‐β, IL‐6, IFN‐γ and TNF‐α significantly decreased over time (Figure S3C), suggesting they were mainly produced early by activated DC, which become less activated towards the end of the culture. In parallel, fresh Treg from the sister were polyclonally stimulated with anti‐CD3/CD28 in the presence of IL‐2, IL‐15 and rapamycin, but without DC. We observed that the levels of IFN‐γ (*p* = 0.00049) and IL‐10 (*p* = 0.00391) were higher in polyclonal Treg than mTreg. IL‐10 was, in fact, below the detection threshold from Day 9 onwards on mTreg cultures, indicating that IL‐10 is not produced by mTreg.

### 2.3 Activation, Proliferation and Metabolic Pathways Were Enriched in Differentially Expressed Genes of mTreg

Transcriptomic analysis revealed differentially expressed genes between Treg from Day 0 (polyclonal, fresh Treg) and mTreg harvested after the selection culture (Day 14) (Figure S6A). In Principal Component Analysis (PCA), the two groups clustered separately, and there was more homogeneity across samples from the mTreg group than from fresh Treg (Figure S6B). mTreg cells presented 2473 differentially expressed genes (1175 up‐regulated and 1298 down‐regulated; log2FC > 0.585, adj. *p* < 0.05), compared to the control of fresh Treg (Table S2). mTreg upregulated several genes encoding molecules involved in lipid biosynthesis (*FASN*, *SQLE*, *ACACA* and *DHCR7*), fatty acid desaturation (*FADS1*, *FADS2* and *SCD*) and cell cycle regulation (*E2F2*, *CDCA7*, *CDK6* and *NEK6*), in accordance with the proliferation mediated by activation of those cells (Figure 3A). Consequently, several biological processes associated with alterations in metabolism or cell activation were significantly enriched within the mTreg upregulated gene set (Figure 3B), such as ‘lipid metabolic process’ (GO:0006629), ‘protein metabolic process’ (GO:0019538), ‘cell activation’ (GO:0001775) and ‘leukocyte activation’ (GO:0045321). Complete network maps are depicted in Figure S7. Corroborating with these data, the Kyoto Encyclopedia of Genes and Genomes (KEGG) terms ‘cell cycle’ (KEGG:04110), ‘metabolic pathways’ (KEGG:01100) and ‘fatty acid metabolism’ (KEGG:01212) were also enriched (Table S3).

Notably, genes coding transcription factors associated with Treg activity, such as *EGR1*, *MYB* and *IRF4* were up‐regulated in mTreg, as well as genes related to cell migration to inflammatory sites (*CCR4*, *CXCR1*, *ITGA3*, *MELK*), the STAT5 target gene *SOCS2* and *CYP1A1*, which has been reported to regulate Treg function and stability [29] (Figure 3A). On the other hand, genes associated with pro‐inflammatory cytokine production (*IFIT1*, *IFIT2*, *IFIT3*, *OASL* and *IRF1‐AS1*) and cAMP cleavage (*PDE4D*, *PDE4DIP* and *PDE4B*) were down‐regulated (Figure 3A). Within the down‐regulated gene sets, there was a significant enrichment in pathways related to RIG‐I signalling, regulation of IFN‐α production and response to TNF (Figure 3C and Table S4), which indicates lower sensitivity of the mTreg to pro‐inflammatory cytokine signalling.

Although some genes associated with tissue‐resident Treg [30] were significantly up‐regulated in mTreg, such as *PDGFA*, *HDAC1* and *KAT2B*, no clear pattern indicative of a specific tissue‐resident Treg subset was observed (Figure 3D). Conversely, several genes linked to a more effector Treg‐like and functional activity were upregulated, including *IL2RA*, *HAVCR2* (TIM3), *LGALS9*, *TNFRSF10B* (TRAILR2), *GZMA* and *TNFRSF1B* (Figure 3D). Overall, these findings demonstrate that the transcriptional changes correlated with the activation, proliferation and augmented function of mTreg upon the HLA‐matched stimuli provided by the DC in the selection culture.

### 2.4 mTreg‐Specific Suppressive Function Relies on Cellular Contact and HLA Class II

**FIGURE 4:**
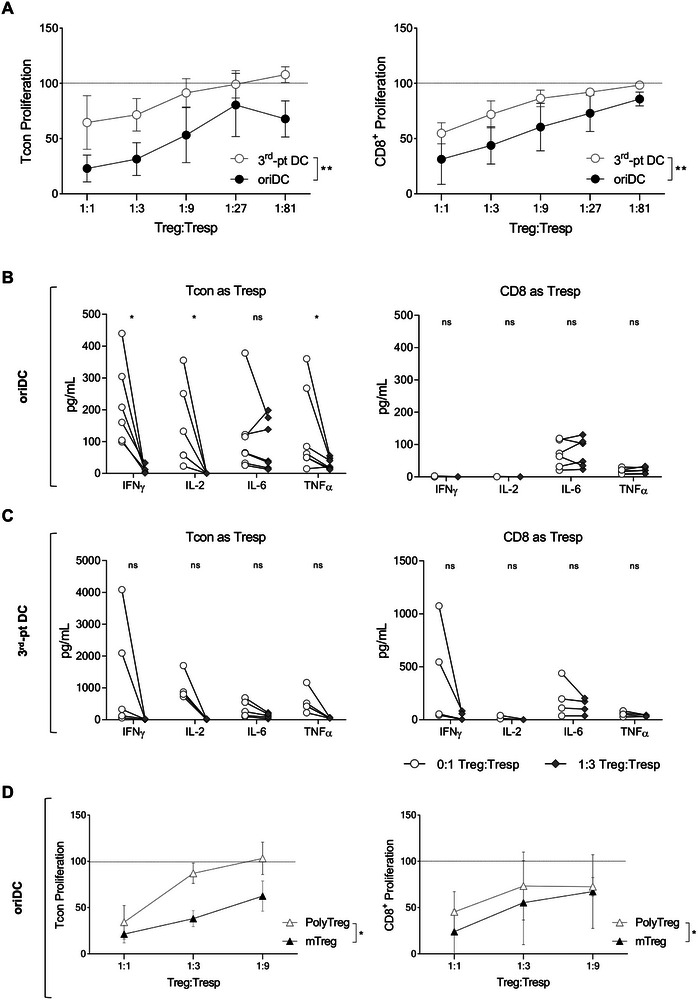
Potent and specific suppression of T cell proliferation and pro‐inflammatory cytokine production by mTreg. (A) SA were performed by assessing the proliferation of the female sibling Tcon (left panel) or CD8^+^ (right panel) as Tresp, at different Treg:Tresp ratios, in response to the brother's HLA‐matched DC (original DC used in the selection culture: oriDC) or to DC from a fully HLA‐mismatched third‐party male donor (third‐pt DC) for 6 days. (B, C) Concentration in pg/mL of IFN‐γ, IL‐2, IL‐6, and TNF‐α in the supernatant of the SA when Tcon (left panel) or CD8^+^ cells (right panel) were used as Tresp at 1:3 Treg:Tresp ratio with oriDC (B), or third‐pt DC (C) as stimuli. Symbols represent each independent experiment. Five to seven experiments are depicted. (D) SA comparing mTreg to polyclonally stimulated Treg from the same female sibling expanded using anti‐CD3/anti‐CD28‐based stimuli. Tcon (left) or CD8^+^ (right) proliferation was assessed in the presence of the brother's HLA‐matched oriDC after 6 days. (A, D) Tresp proliferation was calculated by normalising the frequency of CTV^low^ cells in the presence of Treg to the frequency measured in the absence of Treg, represented by the dotted line at 100 on the *Y*‐axis. Mean + SD of three (A) or two to three (D) independent experiments with independent pairs of siblings is shown. Paired *t*‐tests performed between oriDC versus third‐pt DC groups (A), 0:1 versus 1:3 groups (B, C), and Poly Treg versus mTreg groups (D) *p < 0.05; **p < 0.01; ns, statistically not significant.

In order to determine the potency and specificity of mTreg, we performed in vitro suppression assays (SA) on Day 14. mTreg were co‐cultured with responder T cells (Tresp), that is, Tcon or CD8 T cells, in the presence of the original fully HLA‐matched DC used for selection culture (oriDC) or a third‐party DC from a fully mismatched male donor (third‐pt DC). Our protocol generated highly suppressive mTreg capable of inhibiting both Tcon and CD8 proliferation even at lower Treg:Tresp ratios, more efficiently when the same stimuli of the selection (oriDC) were used rather than the mismatched stimuli (Figure 4A). Moreover, multiplex assays show that mTreg decreased the levels of IFN‐γ, IL‐2 and TNF‐α in the supernatant when Tcon were cultured with oriDC (Figure 4B), but not with third‐pt DC (Figure 4C). We next compared the suppressive capacity of mTreg to polyclonal Treg from the same donor, which were obtained after in vitro culture of freshly isolated Treg with anti‐CD3/CD28 stimuli. SA showed that mTreg were more suppressive than polyclonal Treg on both Tresp proliferation upon HLA‐matched stimuli (Figure D).

**FIGURE 5:**
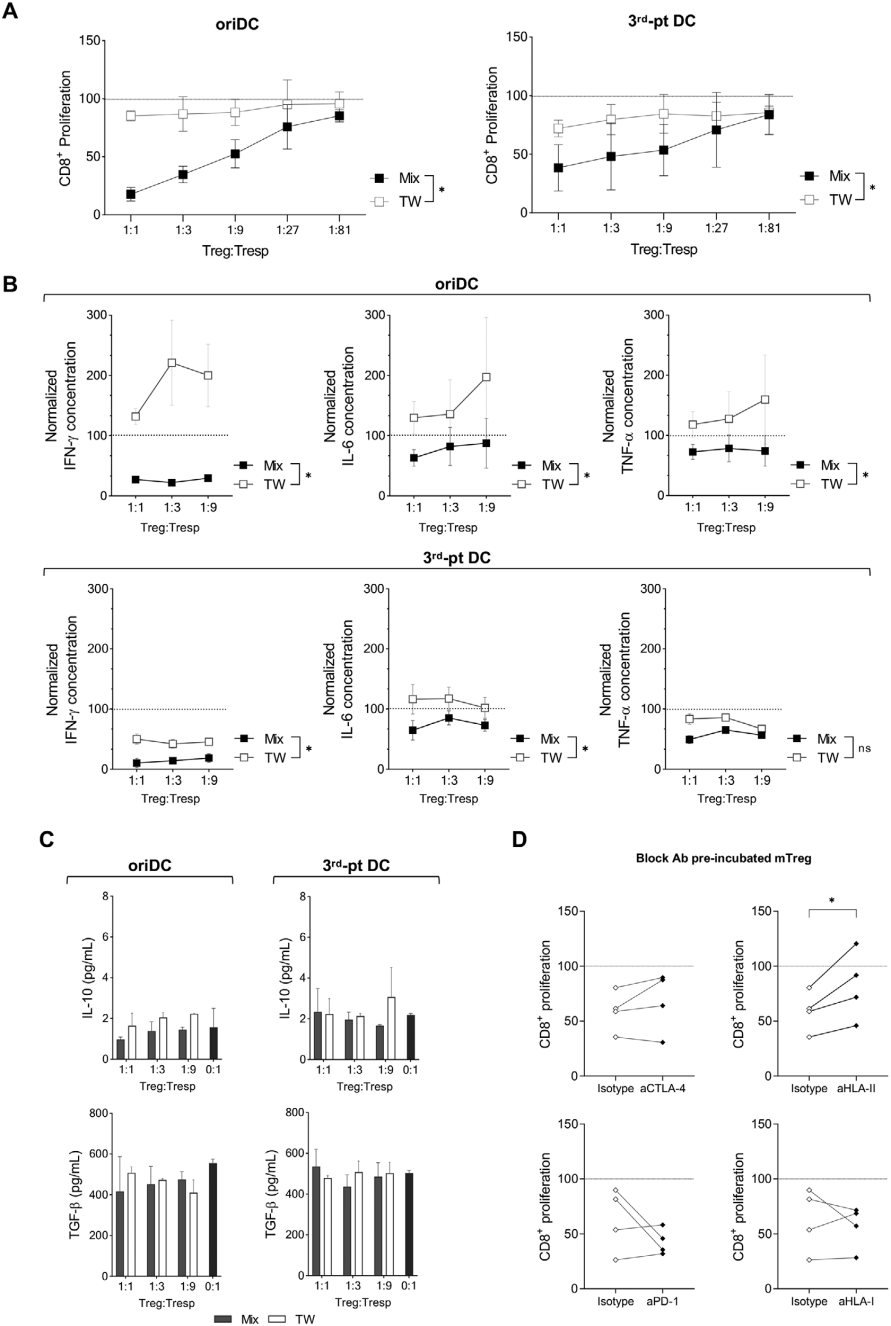
mTreg suppressive function is dependent on cellular contact and involves HLA class II expression on Treg surface. (A) Transwell (TW) SA were performed for 6 days using inserts that physically separate Treg from the Tresp, preventing cell‐to‐cell contact, whilst allowing the exchange of media and soluble factors. Both top and bottom compartments had DC as stimuli, either original HLA‐matched DC from the brother (oriDC, left panel) or third‐party male HLA‐mismatched DC (third‐pt DC, right panel). In parallel, regular SA were conducted in which Treg, Tresp and DC were seeded in the lower compartment, while the insert only contained cell culture media (Mix). CD8^+^ proliferation was calculated by normalising the frequency of CTV^low^ cells in the presence of Treg to the frequency found in the absence of Treg, represented by the dotted line at 100 on the *Y*‐axis. (B) Normalised concentrations of IFN‐γ, IL‐6, and TNF‐α Treg to the quantity measured in the absence of Treg in the TW or Mix supernatants after 6 days of culture with different ratios of Treg:Tresp (CD8^+^ T cells), using either oriDC (top panel) or third‐pt DC (bottom panel) as stimuli. (C) Concentration in pg/mL of IL‐10 and TGF‐β in the supernatant of TW or Mix conditions with CD8^+^ cells as Tresp and either oriDC (left) or third‐pt DC (right) as stimuli. Mean + SD of three (A) or two (B, C) independent experiments using different pairs of siblings are shown. (D) mTreg were pre‐incubated with anti‐CTLA‐4, anti‐PD‐1, anti‐HLA class I (aHLA‐I) or anti‐HLA class II (aHLA‐II) blocking antibodies before being used in a regular SA of 1:3 Treg:CD8 ratio in the presence of oriDC as stimuli. Four independent experiments using different pairs of siblings are depicted. Paired *t*‐tests were performed between Mix versus TW groups (A–C) and between isotype and blocking antibodies (D); Wilcoxon was done in the case of Mix versus TW for IL‐10 in (C); and Kruskal–Wallis was performed between ratios in (C). *p < 0.05; ns, statistically not significant.

As Treg can exert their suppressive function through contact‐dependent and ‐independent mechanisms [31, 32], we sought to investigate the mechanisms involved in mTreg‐mediated suppression. To determine if cellular contact with responders is required, SA were performed using Transwell (TW) inserts to isolate the Tresp from mTreg and compared to conventional SA in which cells are mixed (Mix) in the same well. We observed that mTreg‐mediated suppression of CD8 proliferation (Figure 5A) and pro‐inflammatory cytokine production (Figure 5B) was abrogated in the TW condition, mainly when oriDC were the stimuli. The observation that cytokine production was higher in TW co‐cultures than in their control condition (0:1 Treg:Tresp ratio, represented by the dashed line at 100 on the *Y*‐axis) suggests that the isolation of mTreg in the TW system resulted in increased cytokine production. Thus, Treg‐to‐Tresp contact is required for mTreg function, while soluble factors appear not to play a key role.

Corroborating this idea, in the TW system, there was no difference in IL‐10 and TGF‐β levels in the supernatant with or without mTreg, regardless of the DC used (Figure 5C). Together with the results of cytokine production during the selection culture (Figure S3C), these data suggest that mTreg are not IL‐10 and TGF‐β producers under the conditions tested. Moreover, in SA using polyclonal Treg, we observed that these culture supernatants contained detectable IL‐10 levels while those from mTreg conditions did not (Figure S8). Taken together, these observations indicate that soluble factors are not the main players in mTreg‐specific function.

To further clarify which surface molecules were involved in this contact‐dependent suppression, we pre‐incubated mTreg with blocking antibodies against PD‐1, CTLA‐4, HLA class I (HLA‐I) or HLA class II (HLA‐II) before the SA setup. We observed that PD‐1, CTLA‐4 or HLA‐I blocking on mTreg did not significantly impact their function when CD8 T cells were used as Tresp (Figure 5D), and none interfered when Tresp were Tcon (Figure S9). However, CD8 T cell proliferation with oriDC was higher when anti‐HLA‐II pre‐incubated mTreg were used compared to the isotype control (Figure 5D). Thus, HLA‐II expression on the surface of mTreg appears to have a role in conferring their antigen‐specific suppressive function.

## 3 Discussion

Based on previous work from our group [33], we were able to optimise a protocol for selecting human Treg specific against mHA from an HLA‐matched sibling. Following DC differentiation from the brother's monocytes and subsequent activation, mature DC were typically CD14^−^CD11c^+^CD86^hi^HLADR^hi^CD80^+^CD83^+^, as described previously [34]. This phenotype is associated with higher ability to present antigens, which we believe is particularly important in our setting because alloreactivity will not be driven by a broad HLA library but by minimal differences at the level of mHA. To avoid the expansion of contaminant Tcon, we purified Treg by FACS and supplemented the media with rapamycin [33, 35]. Also, we used IL‐15 in the selection culture, as this cytokine promotes survival, increased Foxp3 levels and sustained suppressive function of Treg [36], as well as maximising proliferation when using rapamycin [37]. We were able to use low‐dose IL‐2 due to the presence of mature DC and IL‐15 [34]. Importantly, mTreg selection culture was performed in the absence of serum to avoid lot‐to‐lot variability and other adverse risks. Serum‐free media not only facilitates the translation of these protocols into the clinical setting but may also contribute to the suppressive stability of mTreg [38]. The co‐culture of fresh Treg with HLA‐matched DC in these conditions led to the proliferation and activation of mTreg, expressing the functional markers PD‐1, CD39, HLA‐DR and CTLA‐4. Importantly, high levels of Foxp3 expression were also sustained, which is imperative as this transcription factor is tightly associated with suppressive capacity and stability of the Treg phenotype.

Corroborating with Foxp3 stability, key regulators of *FOXP3* gene transcription were differentially expressed in mTreg compared to fresh Treg [39, 40, 41, 42]. mTreg highly upregulated *EGR1*, a positive regulator of Foxp3 [39] and downregulated *SIRT1*, a negative regulator of Treg function via Foxp3 deacetylation [43], as well as *RORA* and *HIF1A*, which are involved in fate bifurcation between Treg and Th17 subsets by favouring Th17 differentiation [44].

Metabolic regulation emerged as an important theme among the differentially expressed genes in mTreg. Treg metabolism differs from that of Tcon and is essential for their phenotype, function and proliferation. Recent work has shown that Tregs rely on unsaturated fatty acid synthesis for their proliferation and suppressive function [45]. We observed the enrichment in several lipid pathways and the up‐regulation of genes responsible for fatty acid synthesis and desaturation in mTreg, consistent with the high suppressive function of mTreg.

We have demonstrated that mTreg are endowed with specific suppressive activity, such that HLA‐matched T cell responses were more effectively suppressed than third‐party HLA‐mismatched responses. Cellular contact of mTreg with CD8 responders was necessary for mHA‐specific suppression. Given that the levels of IL‐10 and TGF‐β in supernatants of conditions containing mTreg are similar to those observed in the absence of mTreg, it is likely that those cytokines are not critical for the suppressive activity of mTreg, at least in vitro.

In order to identify which surface molecules could be involved in contact‐dependent mTreg suppressive activity, we performed SA using mTreg pre‐incubated with blocking antibodies. The suppressive ability of mTreg was significantly reduced when HLA‐II molecules on mTreg surface were blocked compared to the isotype control. Activated human Treg expressing HLA‐II molecules have been reported to exert early contact‐dependent suppression [46], which is abrogated by blocking HLA‐II molecules [47]. Moreover, HLA‐DR^+^ Treg are more suppressive than their counterparts, and the expression levels of HLA‐DR positively correlate with Foxp3 [46, 48].

The exact role of HLA‐II on Treg‐mediated suppression remains to be elucidated, and it might not even be mediated by classical HLA functions given that Treg do not carry antigen‐processing machinery [32]. Some of the hypotheses are that HLA‐II (i) engages cytotoxicity [49]; (ii) functions simply to connect Treg with effector T cells, then allowing another mode of suppression to occur or (iii) works as a self‐regulating mechanism involving interaction with Lag‐3 [32]. Our work contributes to this subject by showing that HLA‐II expression on mTreg is associated with suppression dependent on the cellular contact between those cells and CD8 T cells, which indicates that it is indeed probably not a case of classical antigen presentation mediated by the HLA‐II^+^ mTreg. Also, Treg are only pre‐incubated with the blocking antibody, and no more blocking antibody is added during the culture, so its binding to Treg is not likely to persist long into the culture, meaning that it likely has an early effect, as already described [46]. Moreover, when third‐pt DC were used as stimuli, blocking HLA‐II on mTreg did not exert any effect (data not shown). Therefore, our data corroborate that HLA class II‐driven suppression mechanism is a hallmark of the specific Treg function.

In accordance with other studies [22, 23, 24, 34, 50], we have shown that mHA‐specific Treg are more efficient suppressors than polyclonal Treg, being a more suitable option for cGVHD treatment. Current strategies include the development of protocols to obtain alloantigen‐specific Treg [50], which do not fit the HLA‐matched allo‐HSCT scenario, or monoclonal‐specific Treg [25, 26, 27]. Mouse Treg specific against the group of mHA named H‐Y have been induced in vitro [25], and H‐Y‐specific human Treg have been expanded in vitro [51], but these H‐Y‐specific monoclonal iTreg have been reported to have limited potential for clinical translation [52]. H‐Y are mHA only expressed in males—‘H’ represents ‘histocompatibility’ and ‘Y’ refers to the fact that the minor H antigen is encoded by a gene on the Y chromosome, which creates a higher risk for GVHD in HLA‐matched allo‐HSCT with a male recipient and a female donor. On the other hand, there is a reduced risk of relapse in those recipients [53]. Thus, as an mHA known for its association with both GVHD and GVL responses, H‐Y is not an ideal choice for the generation of antigen‐specific Treg.

Recent studies have explored the use of chimeric antigen receptors (CAR) to generate antigen‐specific T cells. In fact, when used in combination with allo‐HSCT, CAR‐T cells have been demonstrating potential to improve treatment outcomes for certain haematological malignancies [54], such as relapsed or refractory aggressive B cell lymphomas. In this line, studies are emerging testing CAR‐Treg cells for promoting transplant tolerance [55]. The adoptive transfer of CAR‐Treg holds promise in suppressing GVHD, while preserving an adequate antitumour immune response [26, 27, 56]. For instance, CAR‐Treg have been developed against one of the most common mismatched antigens, the HLA class I antigen HLA‐A2 [26]. However, HLA class I are broadly expressed, which may result in robust CAR stimulation and create a generalised immunosuppressive state that could impair GVL response. Moreover, CAR‐Treg are able to employ some level of antigen‐specific cytotoxicity despite CAR‐specificity [57], which again could be a problem in such ubiquitous antigens. Therefore, we believe that our protocol, which is designed for the enrichment of donor Treg specific against an array of mHA in the recipient's cells, will be capable of providing more efficient and direct suppression of GVHD, likely sparing the GVL effect, by being more likely to target mHA that are involved in the disease but not in tumour responses.

### 3.1 Data Limitations and Perspectives

The aim of this work was to generate and characterise mTreg specific to a range of mHA, which is personalised between each allo‐HSCT donor/recipient pair. Thus, the use of tetramers or similar techniques to identify the mTreg would not be suitable in this context, as it would require precise knowledge of the target antigens. TCR sequencing would primarily reveal a reduction in clonal diversity between the initial fresh, polyclonal Treg and the post‐selection mTreg population and can be performed in future studies; however, the critical question addressed hereby was whether the selected clones were suppressive in an antigen‐specific manner. Regarding suppressive mechanisms, although not explored in this work, follow‐up studies could benefit from our transcriptomic data to investigate the roles of intracellular cAMP [58], granzyme A [59] and galectin‐9 [60] in mTreg‐mediated suppression.

Looking towards clinical translation, we are now seeking the development of a robust and consistent manufacturing process for our protocol, using GMP‐grade reagents and facilities, as well as including an expansion step to increase Treg numbers for future infusions. Nevertheless, the required number of antigen‐specific Treg will likely be lower than for polyclonal Treg [61, 62]. Ultimately, in addition to determining the cell dose needed, it will be important to assess that GVL will, in fact, not be affected by mTreg [63]. Overall, we believe that our findings pave the way for the use of mTreg obtained using this protocol to treat cGVHD using a cellular product that is tailored to the needs of each patient.

## 4 Materials and Methods

### 4.1 Cell Source and HLA Typing

Approximately 100 mL of peripheral blood was obtained from 43 healthy volunteer pairs of siblings from the opposite biological sex. PBMC were isolated by Ficoll density gradient separation. The study was approved by the Ethics Committee of the Lisbon Academic Medical Centre (No. 470/20) and all participants provided written informed consent. HLA typing was performed by high‐resolution genotyping at Instituto Português do Sangue e Transplantação (Lisbon). Fully HLA‐matched pairs of siblings (paired for HLA‐A, ‐B, ‐C class I and HLA‐DRB1, ‐DQA1, ‐DQB1 loci) were selected. Third‐party male donors who were 100% HLA‐mismatched to the fully HLA‐matched pair of siblings were used as controls.

### 4.2 Flow Cytometry

The anti‐human monoclonal antibodies used for flow cytometry analysis are listed in the supplemental Table S5. Fixable Viability Dye e506 (eBioscience) was used to identify live cells. Intracellular staining was performed using Foxp3/Transcription Factor Fixation/Permeabilisation reagents (eBioscience) and the monoclonal antibody FOXP3‐e450 (PCH101, eBioscience). Samples were run on BD LSRFortessa X‐20 (BD Biosciences) or LSRFortessa (BD Biosciences). Data were analysed using FlowJo v10.8 (BD Biosciences) and its Proliferation Modelling plugin [64].

### 4.3 Differentiation of moDC

CD14^+^ monocytes were isolated from sibling or third‐party male PBMC with EasySep Human CD14 Positive Selection Kit II (STEMCELL Technologies) and cultured in X‐VIVO 15 supplemented with 100 U/mL penicillin/100 µg/mL streptomycin (Gibco), recombinant human IL‐4 (40 ng/mL) and GM‐CSF (50 ng/mL) from PeproTech at 37°C, 5% CO_2_ for 5 days. Fresh medium, IL‐4 and GM‐CSF were added on Day 3. Next, differentiated moDC were activated with IL‐1β (10 ng/mL), IL‐6 (10 ng/mL), TNF‐α (20 ng/mL) from PeproTech, and PGE_2_ (1 µg/mL, Tocris Bioscience) for 24 h. Activated moDC were then cryopreserved until their use on mTreg selection cultures or SAs, when they were thawed and re‐activated with the same cytokines for additional 20 h.

### 4.4 In Vitro Selection of mTreg

CD4 T cells from female siblings were isolated by negative selection from PBMC using EasySep Human CD4 T Cell Isolation Kit (STEMCELL Technologies) and rested at 37°C, 5% CO_2_ overnight, while the CD4‐negative fraction was cryopreserved for future use. Treg (CD3^+^CD4^+^CD25^high^CD127^low^) and Tcon (CD3^+^CD4^+^CD25^−^) were sorted from CD4 T cells on BD FACSAria Fusion (BD Biosciences). Purity was > 98% for both populations. Sorted Tcon were cryopreserved for future use in SAs. Treg were cocultured with γ‐irradiated (30 Gy) fully HLA‐matched moDC at a Treg:DC ratio of 1:1 (10^5^ or 5 × 10^4^ each) or 4:1 (8 × 10^4^ Treg and 2 × 10^4^ DC) in round‐bottom 96‐well plates using xeno‐ and serum‐free culture conditions: TexMACS medium supplemented with 100 U/mL penicillin/100 µg/mL streptomycin (Gibco), recombinant human IL‐2 (10 U/mL, R&D Systems), IL‐15 (10 ng/mL, R&D Systems) and rapamycin (100 ng/mL, Calbiochem/Sigma‐Aldrich). From Day 7 onwards, medium was replenished every 2 days with TexMACS supplemented with IL‐2 (10 U/mL), until Day 14. To access cell proliferation, CD4 T cells were labelled with 0.5 µM CFSE Proliferation Kit (Invitrogen) at 37°C, 5% CO_2_ for 15 min before the co‐culture. As a polyclonal stimulus control, fresh Treg cells from the same female sibling donor were cultured with ImmunoCult Human CD3/CD28 T Cell Activator (STEMCELL Technologies), using the same conditions of mTreg culture but without the addition of DC. As a control, CD3^+^CD4^+^CD25^−^ Tcon from the same female sibling were cultured with HLA‐matched DC from the respective brother in parallel with Treg, but without rapamycin. Treg fold expansion was calculated by dividing the total number of cells counted after co‐culture by the number of Treg seeded on Day 0.

### 4.5 Suppression Assays

One day before the SA, female sibling cryopreserved Tcon and CD4‐negative T cells were thawed and incubated with DNase (0.1 mg/mL, Roche) for 15 min and rested overnight at 37°C, 5% CO_2_. CD8 T cells were then sorted from the CD4‐negative fraction on BD FACSAria Fusion (BD Biosciences). Tcon or CD8 T cells were labelled with 2.5 µM CellTrace Violet (CTV) Cell Proliferation Kit (Invitrogen) at 37°C, 5% CO_2_ for 20 min to be used as Tresp. mTreg were titrated to 1:1, 1:3, 1:9, 1:27, and 1:81 ratios of Treg:Tresp in duplicate on 96‐well plates with 25 × 10^3^ Tresp and 25 × 10^3^ γ‐irradiated (30 Gy) moDC from the fully HLA‐matched male sibling (oriDC) or an HLA‐mismatched third‐party male donor (third‐pt DC control). Wells without mTreg (0:1 Treg:Tresp ratio) were used as negative controls. Cells were incubated for 6 days in RPMI 1640 with 10% heat‐inactivated human AB serum (Sigma‐Aldrich), 2 mM l‐glutamine (Gibco), and 100 U/mL penicillin/100 µg/mL streptomycin (Gibco). Tresp were identified as CD3^+^CD8^+^ or CD3^+^CD4^+^. To exclude mTreg from the Tcon analysis, a gate selecting CD4^+^CTV‐labelled cells was used, excluding CTV‐negative cells by comparison with wells containing only Treg or only Tcon. Tresp proliferation was calculated by normalising the frequency of CTV^low^ Tresp in the presence of mTreg to the frequency found in the absence of mTreg.

### 4.6 TW SA

CD3^+^CD8^+^ Tresp were obtained from the female sibling donor and labelled with CTV as described above. Tresp were physically separated from mTreg through inserts of a 96‐well Transwell plate (Corning). A total of 25 × 10^3^ Tresp were cultured in duplicate wells in the lower compartment, while mTreg were cultured in the upper compartment at the same concentrations as the standard SA conditions. 25 × 10^3^ γ‐irradiated moDCs from the respective fully HLA‐matched brother (oriDC) or third‐pt DC were added to both compartments. SAs were conducted simultaneously, in which Treg and Tresp were seeded with DC in the lower compartment, respecting the same Treg:Tresp ratios, while the insert contained only the cell culture media. Tresp proliferation was measured in the lower compartments by CTV dilution on Day 6 as described in the SA section.

### 4.7 Antibody Blocking

mTreg were pre‐incubated before the SA with 50 µg/mL of the purified mouse anti‐human antibody anti‐PD‐1, ‐HLA‐A/B/C, ‐CTLA‐4 or ‐HLA‐DR/DP/DQ (all from BD Biosciences) (Table S5) at 4°C for 45 min. Isotype‐matched antibodies were used as specificity controls.

### 4.8 Cytokine Quantification

At the end of selection and suppression cultures, 100 µL of each well supernatant was collected and stored at −20°C for multiplex analysis. Cytokine concentrations were quantified using the MILLIPLEX MAP Human High Sensitivity T Cell Magnetic Bead Panel and the MILLIPLEX MAP TGFß1 Magnetic Bead Single Plex Kit (Millipore). Samples were acquired on a MAGPIX System (Luminex) and analysed using the xPONENT (Luminex).

### 4.9 RNA Extraction and RNA Sequencing

For whole transcriptome profiling by RNA sequencing, we stored in RNA Protect (Qiagen) at −80°C a maximum of 100.000 cells of fresh Treg isolated by FACS before the selection culture (Day 0), as well as mTreg from the end of the selection culture (Day 14), which were FACS‐purified to separate mTreg from DC. Total RNA was isolated using the RNeasy Plus Micro Kit (Qiagen), following the manufacturer's instructions. Concentration assessment and quality control of RNA were conducted on the Fragment Analyzer (Agilent) with the High Sensitivity RNA Analysis Kit. Only samples with RNA Integrity Number (RIN) greater than 8 were selected for subsequent analyses. Full‐length cDNAs were prepared by the SMART‐Seq2 protocol [65]. After quality control using Fragment Analyzer (Agilent), library preparation, including cDNA ‘tagmentation’, PCR‐mediated adaptor addition and amplification, was done following the Nextera library preparation protocol (Nextera XT DNA Library Preparation kit, Illumina) [66]. Libraries were confirmed by Fragment Analyzer (Agilent) and then sequenced (NextSeq2000, Illumina) using 100 SE P2. Illumina DRAGEN FASTQ Generation v3.8.4 was used to obtain FastQ files. Library preparation and sequencing were optimised and performed by the Genomics Platform at the Gulbenkian Institute for Molecular Medicine (GIMM).

### 4.10 Differential Gene Expression Analysis

The FastQ reads were aligned against the human reference genome GRCh38 (Ensembl release 111) using that STAR aligner (STAR; v.2.7.11b). Gene‐level counts were quantified against the Ensembl 111 annotation with FeatureCounts (featureCounts v2.0.2) [67] to perform read summarisation by assigning uniquely mapped reads to genomic features. Differential Gene Expression (DGE) and data visualisation were performed in R (v4.4.2), using the DESeq2 R package [68] (v.1.46.0). Gene expression was modelled by a one‐factor linear model using Treg type as a factor and specified to DESeq2 with the design: counts ∼ Treg type (fresh Treg, *n* = 4 and mTreg, *n* = 4). Genes presenting an average expression inferior to five counts were removed, resulting in 13,842 genes for downstream DGE analysis. DGE threshold was an adjusted *p* < 0.05 and an absolute log2‐fold change > 0.585 (1.5‐fold). Volcano plots were done with the ggplot2 R package (v.3.5.2) and heatmap with the pheatmap R package (v1.0.12). Gene ontology (GO) (biological process) and KEGG analysis were performed using g:Profiler [69] with a significance threshold Benjamini–Hochberg FDR (0.05) and excluding electronic GO annotations. Visualisation of g:Profiler enriched gene sets data was obtained using Cytoscape (v3.10.3) software with the Enrichment Map (v3.5.0) and the AutoAnnotate (v1.5.2) plugins [70]. Edges between gene sets were defined with a Jaccard overlap combined coefficient cutoff of 0.375 used to determine similarity, and the *q* value node cutoff was 0.05.

### 4.11 Statistical Analysis

Statistics were performed using R (version 4.2.2). Statistical significance was set at *p* < 0.05, and a confidence level of 0.95 was used. For comparisons between conditions, Student paired t‐test or Wilcoxon signed‐rank test (between two samples); one‐way ANOVA, Welch one‐way ANOVA or Kruskal–Wallis followed by Dunn's test (for multiple comparisons), were carried out as appropriate, considering the required assumptions of each model. *p < 0.05; **p < 0.01; ***p < 0.001; ****p < 0.0001. Graphs were generated in Prism (v8, GraphPad).

## Author Contributions

C.P. designed experiments, raised the volunteers, conducted experiments, analysed data, conducted statistical analyses and wrote the manuscript. J.L. and R.A. developed the conceptual framework of the study. C.P., M.S. and J.L. discussed the data. L.R. performed the HLA typing technique and analysis. H.L. conducted DGE analysis. M.S., R.A. and J.L. revised the manuscript.

## Conflicts of Interest

The authors declare no conflicts of interest.

## Ethics Statement

The study was approved by the Ethics Committee of the Lisbon Academic Medical Centre (No. 470/20) and all participants provided written informed consent.

## Supporting information




**Supporting File 1**: eji70096‐sup‐0001‐SuppMat.pdf.

**Supporting file 2**: eji70096‐sup‐0002‐TableS2.xlsx

**Supporting file 3**: eji70096‐sup‐0003‐TableS3.xlsx

**Supporting file 4**: eji70096‐sup‐0004‐TableS4.xlsx

## Data Availability

The data that support the findings of this study are available from the corresponding author upon reasonable request: joao.lacerda@gimm.pt.
