# Supplementary material for: Selection and Characterisation of Minor Histocompatibility Antigen‐Specific Regulatory T Cells in Fully HLA‐Matched Setting for GVHD Therapy
Source: Eur J Immunol. 2025 Dec 18;55(12):e70096. doi: 10.1002/eji.70096 (PMC12712890; doi:10.1002/eji.70096)
Supplement: Supplementary file 1 — Supporting File 1: eji70096‐sup‐0001‐SuppMat.pdf. [file EJI-55-e70096-s001.pdf]

SUPPLEMENTAL INFORMATION

Selection and characterisation of minor histocompatibility antigen-specific regulatory T cells in fully HLA-matched setting for GVHD therapy

Carolina P. Pacini,[1] Rita I. Azevedo,[1] Luís Ramalhete,[2,3,4] Hugo A.J. Lainé,[5] Maria V.D. Soares,[1,6] and João F. Lacerda[1,6,7]
[1]JLacerda Lab, Gulbenkian Institute for Molecular Medicine, Lisbon, Portugal; [2]Blood and Transplantation Center of Lisbon, Instituto Português do Sangue e da Transplantação, Lisbon, Portugal; [3]NOVA Medical School, Universidade NOVA de Lisboa, Lisbon, Portugal; [4]iNOVA4Health-Advancing Precision Medicine, Núcleo de Investigação em Doenças Renais, NOVA Medical School, Faculdade de Ciências Médicas, Universidade NOVA de Lisboa, Lisbon, Portugal; [5]Advanced Data Analysis, Gulbenkian Institute for Molecular Medicine, Lisbon, Portugal; [6]Faculdade de Medicina, Universidade de Lisboa, Lisbon, Portugal; and [7]Serviço de Hematologia e Transplantação de Medula, ULS Santa Maria, Lisbon, Portugal

SUPPLEMENTAL FIGURES

- Figure S1: DC were successfully differentiated from monocytes and then activated with a gold-standard cytokine cocktail.
- Figure S2: Gating strategy and purity of T cell sorting.
- Figure S3: Time-course of activation and cytokine secretion of mTreg during fully HLA-matched selection culture.
- Figure S4: Median Fluorescence Intensity (MFI) of activation markers on CD25hiFoxp3hi mTreg during fully HLA-matched selection culture.
- Figure S5: Activation and functional marker expression on mTreg during fully HLA-matched selection culture.
- Figure S6: MA plot and Principal component analysis (PCA) of mTreg RNAseq.
- Figure S7: Complete network of biological pathways within mTreg differentially expressed genes.
- Figure S8: mTreg specific suppression does not rely on IL-10 and TGF-β secretion.
- Figure S9: Suppression of CD4$^+$ Tcon by mTreg in vitro may not depend exclusively on PD-1, CTLA-4, HLA class I or class II interactions.

SUPPLEMENTAL TABLES

- Table S1: High-resolution HLA sequencing from fully HLA-matched siblings of opposite sexes and the third-party (3rd-pt) fully HLA-mismatched donors used to generate the data presented in each figure.
- Table S2: Differentially expressed genes in mTreg. – *attached file*
- Table S3: Enrichment pathways within upregulated genes. – *attached file*
- Table S4: Enrichment pathways within downregulated genes. – *attached file*
- Table S5: List of primary antibodies.

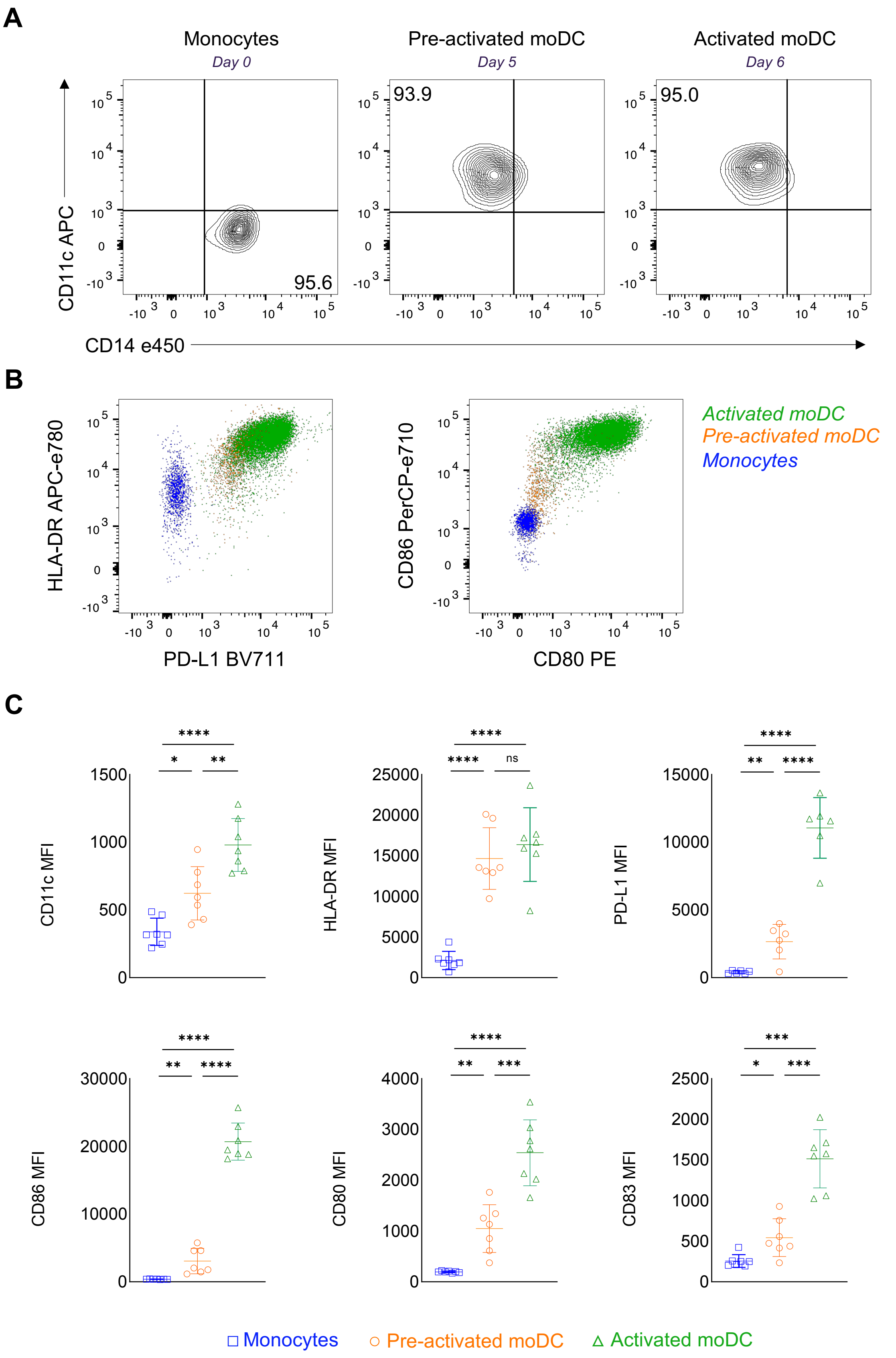
A
Monocytes
Day 0
Pre-activated moDC
Day 5
Activated moDC
Day 6
CD11c APC
CD14 e450
95.6
93.9
95.0
B
HLA-DR APC-e780
PD-L1 BV711
CD86 PerCP-e710
CD80 PE
Activated moDC
Pre-activated moDC
Monocytes
C
CD11c MFI
HLA-DR MFI
PD-L1 MFI
CD86 MFI
CD80 MFI
CD83 MFI
ns
Monocytes
Pre-activated moDC
Activated moDC

Figure S1: DC were successfully differentiated from monocytes and then activated with a gold-standard cytokine cocktail. $CD14^+$ monocytes were isolated from the respective HLA-matched male sibling PBMC and cultured for 5 Days with GM-CSF and IL-4. The monocyte-derived dendritic cells (moDC) generated were then activated with IL-1β, IL-6, TNF-α, and PGE_2 for 24h and cryopreserved until their use for mTreg selection and SA. Representative plots of the expression of CD11c vs. CD14 (A), HLA-DR vs. PD-L1 and CD86 vs. CD80 (B) by monocytes, pre-activated moDC and moDC after 24 hours of activation. After thawing, a 20-hour round of activation was performed with the same cytokines, and the expression of those markers remained the same (data not shown). (C) Median fluorescence intensity (MFI) of each activation marker in monocytes (blue squares) and moDC pre- (orange circles) and post-activation (green triangles). Mean + SD of data obtained from six (PD-L1) to seven (others) independent experiments with different male donors. The statistical models used were Welch one-way ANOVA (for CD80, CD86, HLA-DR, PD-L1), Kruskal-Wallis followed by Dunn's test (for CD83) and one-way ANOVA (for CD11c). *, $p < 0.05$; **, $p < 0.01$; ***, $p < 0.001$; ****; $p < 0.0001$.

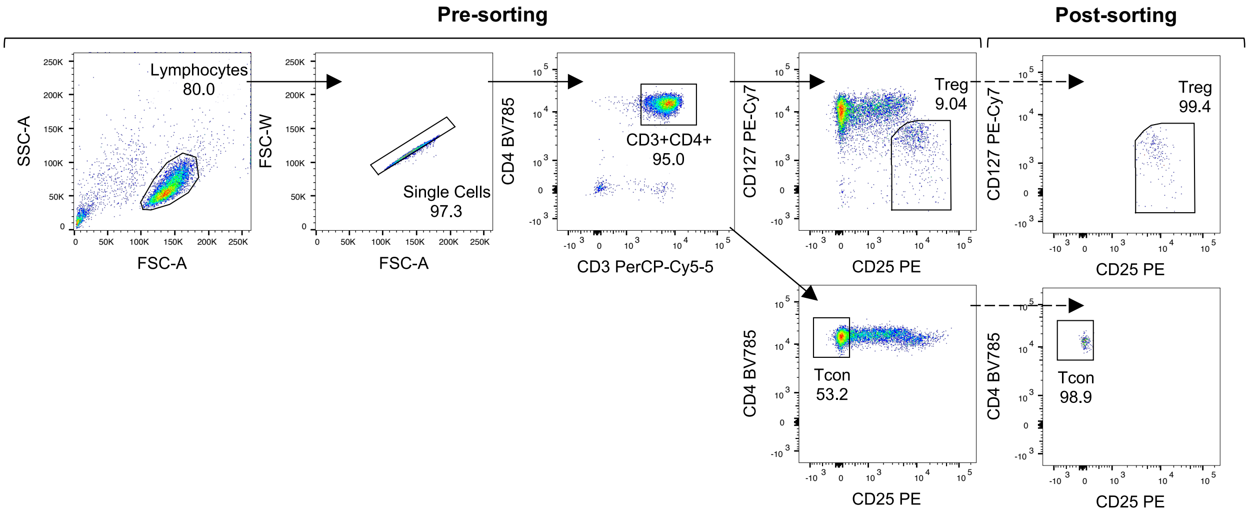

Figure S2: Gating strategy and purity of T cell sorting. $CD4^+$ T cells were negatively selected by immunomagnetic beads from female sibling PBMC and rested overnight. Then, pre-purified $CD4^+$ T cells were counted and stained for CD3, CD4, CD25 and CD127. After gating for the lymphocytes and duplet exclusion, Treg were sorted as $CD3^+CD4^+$ cells with low/negative expression of CD127 and high expression of CD25. In some experiments, $CD3^+CD4^+CD25^-$ were also sorted as conventional T cells (Tcon). Sort purity was above 98% in all experiments.

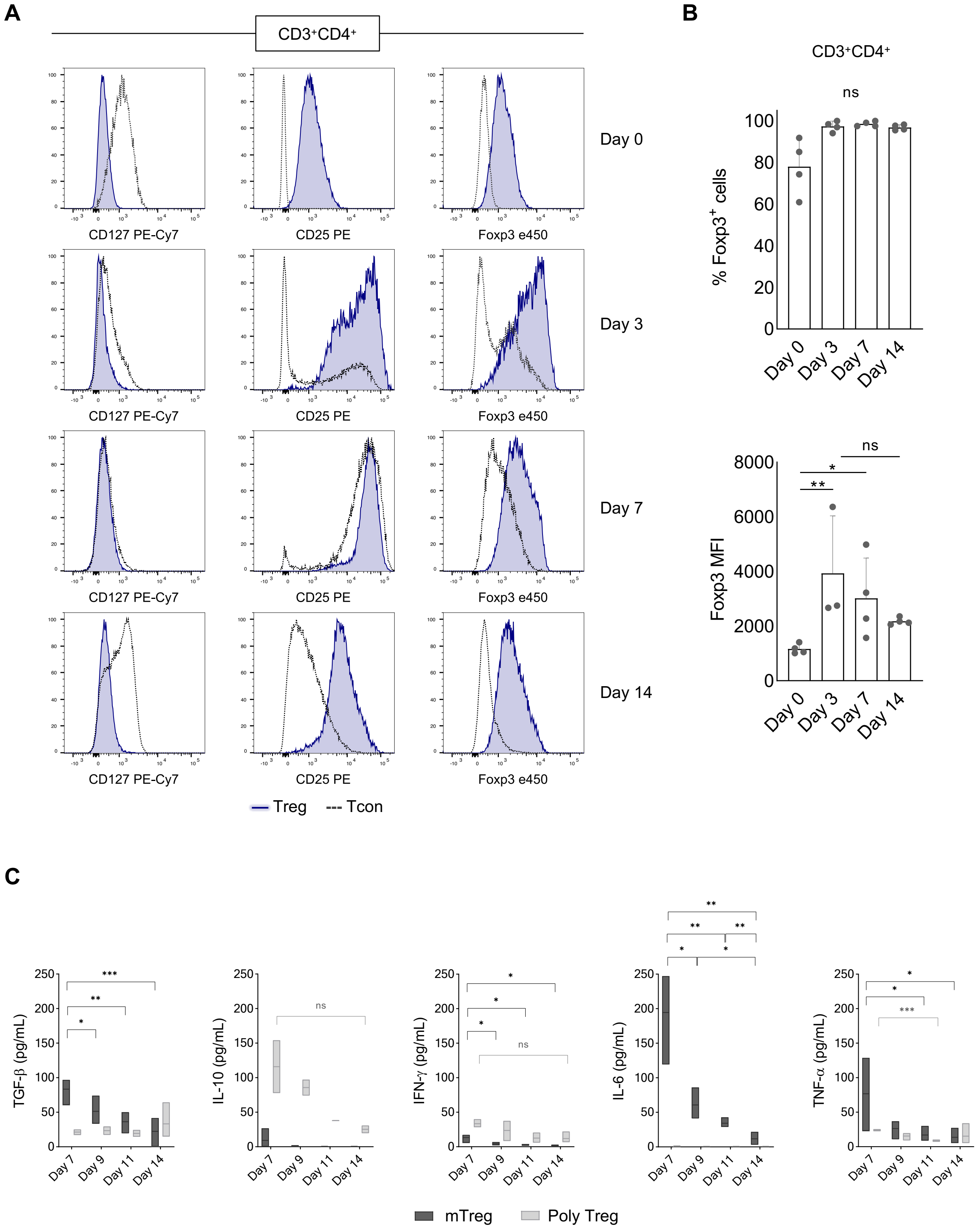
A
CD3⁺CD4⁺
Day 0
Day 3
Day 7
Day 14
CD127 PE-Cy7
CD25 PE
Foxp3 e450
Treg
Tcon
B
CD3⁺CD4⁺
ns
% Foxp3⁺ cells
Foxp3 MFI
Day 0
Day 3
Day 7
Day 14
C
TGF-β (pg/mL)
IL-10 (pg/mL)
IFN-γ (pg/mL)
IL-6 (pg/mL)
TNF-α (pg/mL)
Day 7
Day 9
Day 11
Day 14
mTreg
Poly Treg

Figure S3: Time-course of activation and cytokine secretion of mTreg during fully HLA-matched selection culture. Female sibling Treg (or Tcon as a control) were co-cultured with male sibling HLA-matched DC in a 1:1 Treg:DC $5x10^4$ ratio. CD127, CD25 and Foxp3 expression was assessed on Days 0, 3, 7, or 14 (A) Representative histograms compare each marker expression within the $CD3^+CD4^+$ population in Treg and Tcon. (B) Frequency of Foxp3-positive cells (upper panel) and Foxp3 Median Fluorescence Intensity (MFI) (lower panel) within $CD3^+CD4^+$ cells on Treg selection culture. (C) Cytokine concentration in pg/mL in the supernatant of mTreg selection culture or polyclonal Treg culture at different time points. Mean + SD of three to four (B) or three to eight (C) independent experiments are shown. The statistical models used were Welch one-way ANOVA (for Foxp3, IL-6, TNF-α mTreg, IFN-γ mTreg), Kruskal-Wallis followed by Dunn's test (for IL-10) and one-way ANOVA (for TGF-β, TNF-α Poly Treg, IFN-γ Poly Treg). *, $p<0.05$; **, $p < 0.01$; ***, $p < 0.001$; ns, statistically not significant.

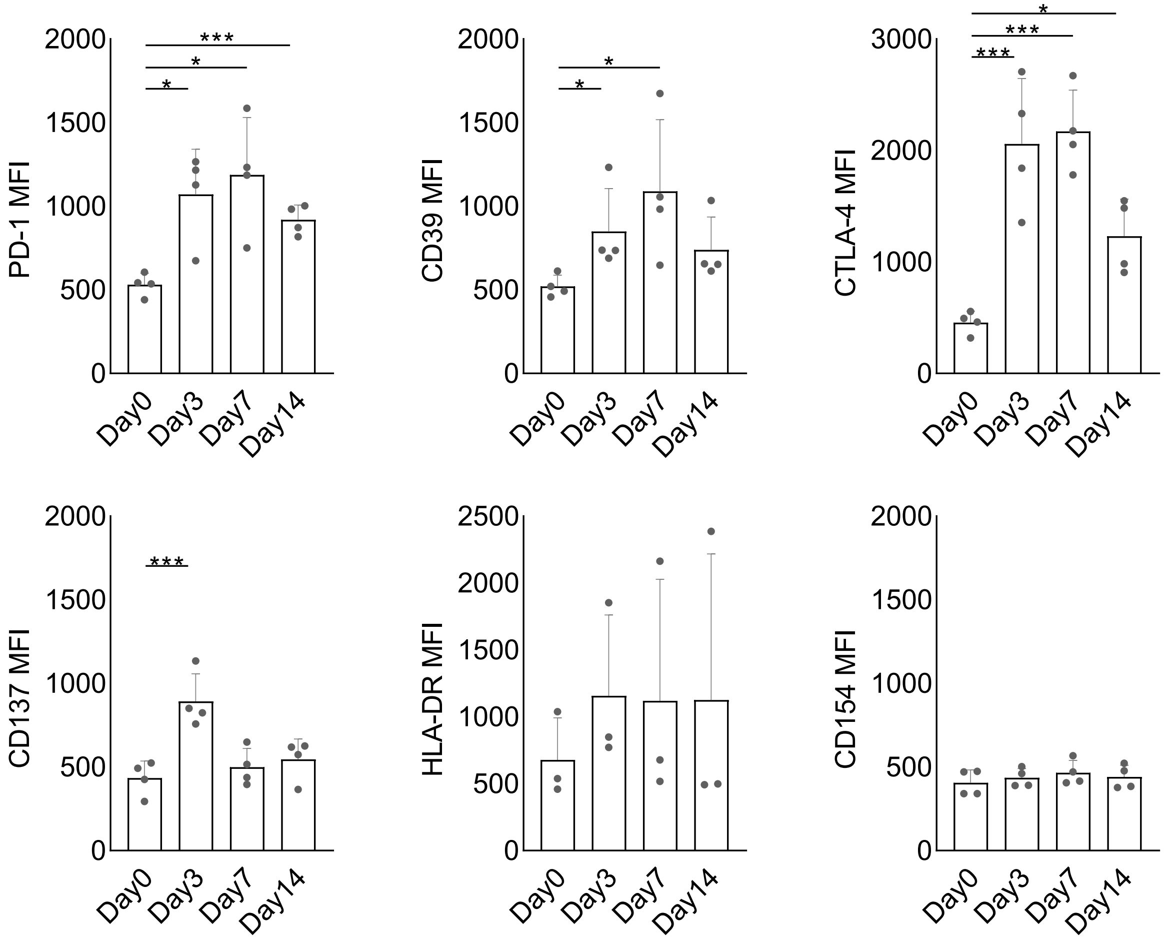

Figure S4: Median Fluorescence Intensity (MFI) of activation markers on CD25^{hi}Foxp3^{hi} mTreg during fully HLA-matched selection culture. Female sibling Treg were co-cultured with male sibling HLA-matched DC at 1:1 Treg:DC 5x10^{4} ratio as described. Phenotyping of CD3^{+}CD4^{+}CD25^{hi}Foxp3^{hi} Treg cells was performed by flow cytometry on Day 0 (pre-selection culture), Day 3, Day 7 and Day 14 of the selection culture, and Median Fluorescence Intensity (MFI) values calculated in FlowJo™ v10.8 (BD Biosciences) in each timepoint are depicted. Mean + SD of three to four independent experiments are shown (bars). Symbols represent each experiment. The statistical models used were Welch one-way ANOVA (for PD-1), Kruskal-Wallis followed by Dunn's test (for CD39, HLA-DR and CD154) and one-way ANOVA (for CD137 and CTLA-4). *, $p<0.05$; ***, $p < 0.001$.

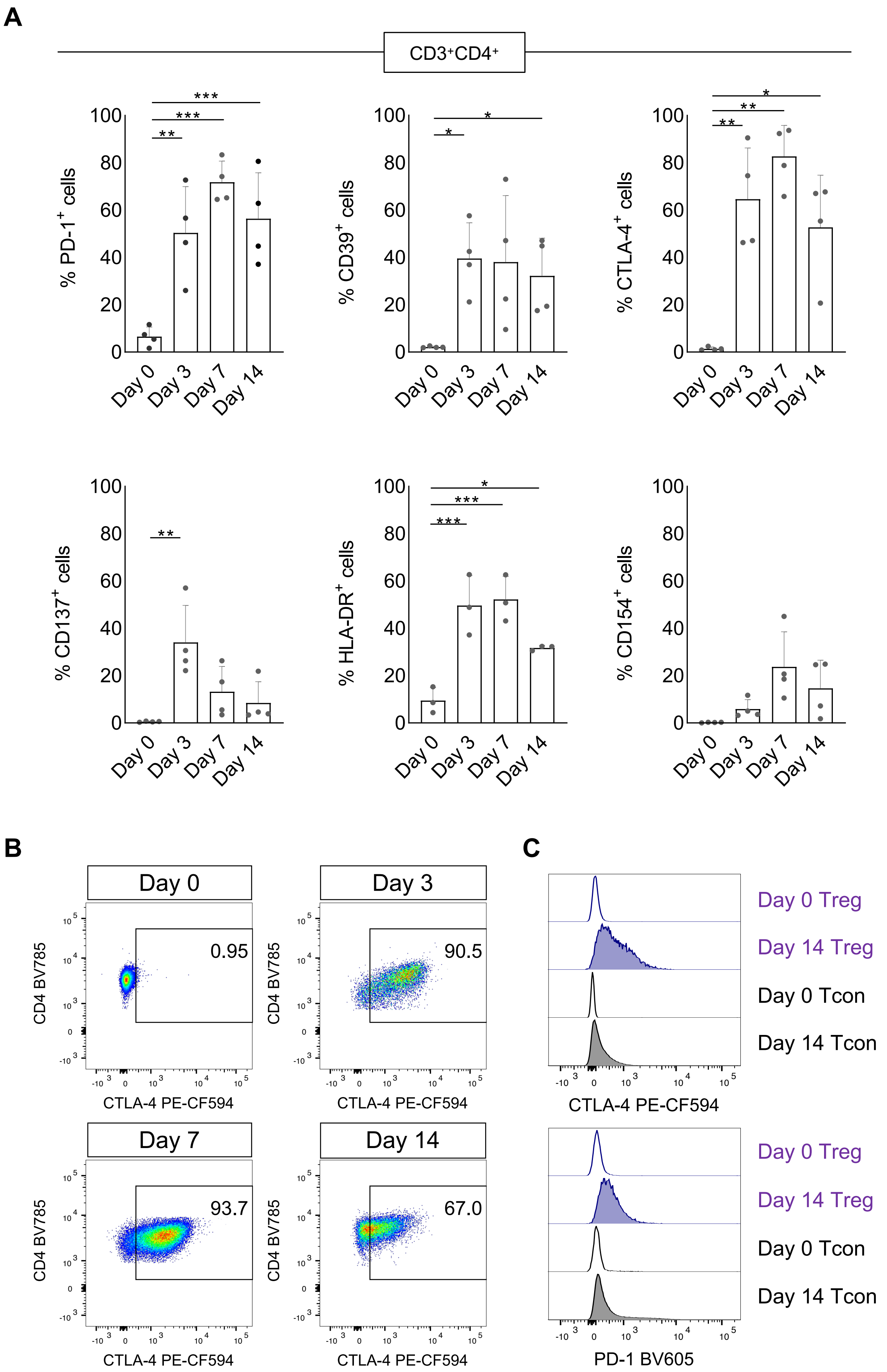
A
CD3+CD4+
% PD-1+ cells
% CD39+ cells
% CTLA-4+ cells
% CD137+ cells
% HLA-DR+ cells
% CD154+ cells
Day 0
Day 3
Day 7
Day 14
B
CD4 BV785
CTLA-4 PE-CF594
0.95
90.5
93.7
67.0
C
Day 0 Treg
Day 14 Treg
Day 0 Tcon
Day 14 Tcon
CTLA-4 PE-CF594
PD-1 BV605

Figure S5: Activation and functional marker expression on mTreg during fully HLA-matched selection culture. Phenotyping of mTreg cells generated as described in Figure 3 considering the $CD3^{+}CD4^{+}$ cells on Days 0, 3, 7 or 14 of the selection culture. (A) Frequency of positive cells for PD-1, CD39, CTLA-4, CD137, HLA-DR and CD154 within the $CD3^{+}CD4^{+}$ population. Each dot represents an independent experiment using different pairs of donors. Mean + SD of data obtained from three to four independent experiments are shown. The statistical models used were Welch one-way ANOVA (for CD39, CD154, CTLA-4), Kruskal-Wallis followed by Dunn's test (for CD137) and one-way ANOVA (for PD-1, HLA-DR). *, $p<0.05$; **, $p<0.01$; ***, $p<0.001$. (B) Representative pseudo-colour plots of $CD3^{+}CD4^{+}CTLA\text{-}4^{+}$ per timepoint. (C) PD-1 and CTLA-4 expression on $CD3^{+}CD4^{+}$ Treg or Tcon on Day 0, and mHA-specific Treg or Tcon on Day 14.

A

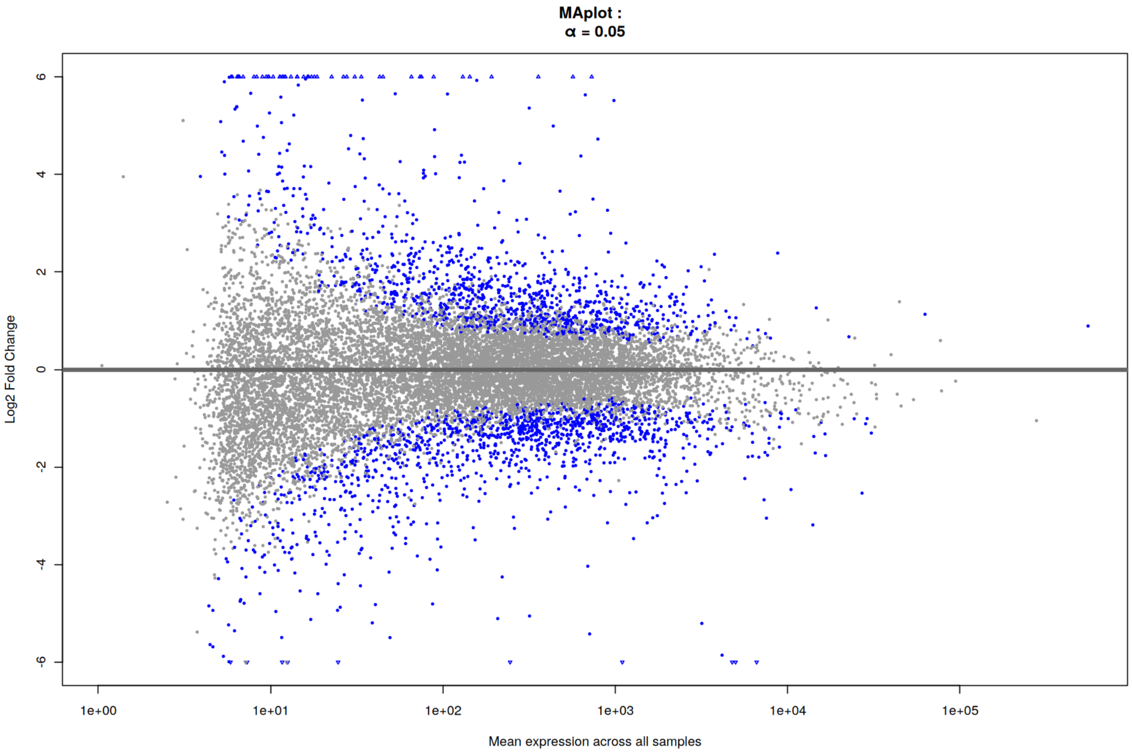
MAplot :
α = 0.05
Log2 Fold Change
6
4
2
0
-2
-4
-6
1e+00
1e+01
1e+02
1e+03
1e+04
1e+05
Mean expression across all samples

B

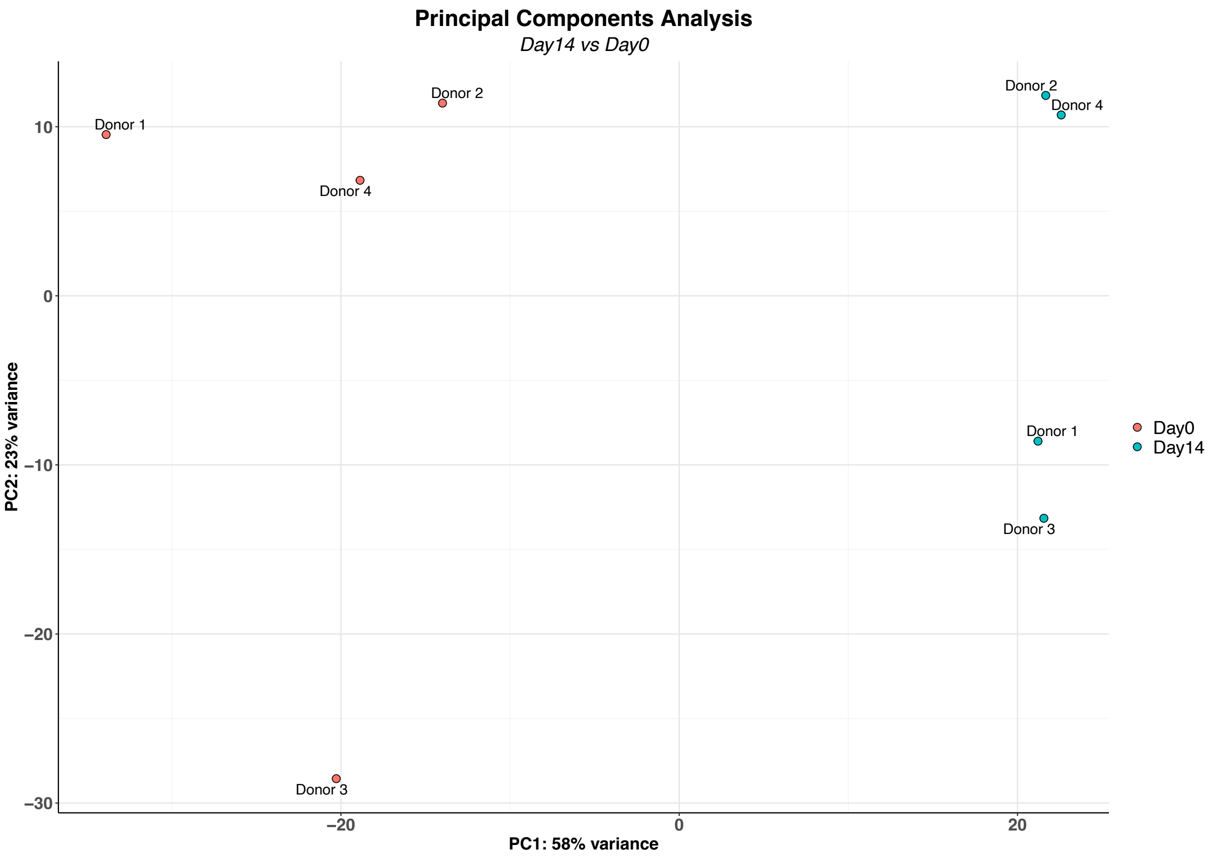
Principal Components Analysis
Day14 vs Day0
PC2: 23% variance
10
0
−10
−20
−30
−20
0
20
PC1: 58% variance
Donor 1
Donor 2
Donor 4
Donor 3
Donor 2
Donor 4
Donor 1
Donor 3
Day0
Day14

Figure S6: MA plot and Principal component analysis (PCA) of mTreg RNAseq. (A) MA plot of differential gene expression compares the log2 Fold-Change (log2FC) to normalised mean counts (the average abundance of gene expression), $\alpha = 0.05$. (B) Overall transcriptional profile of fresh Treg (Day 0), as a control, and mTreg (Day 14), as a case condition, was compared in a PCA analysis, based on the top 500 most variable genes in the data set.

A

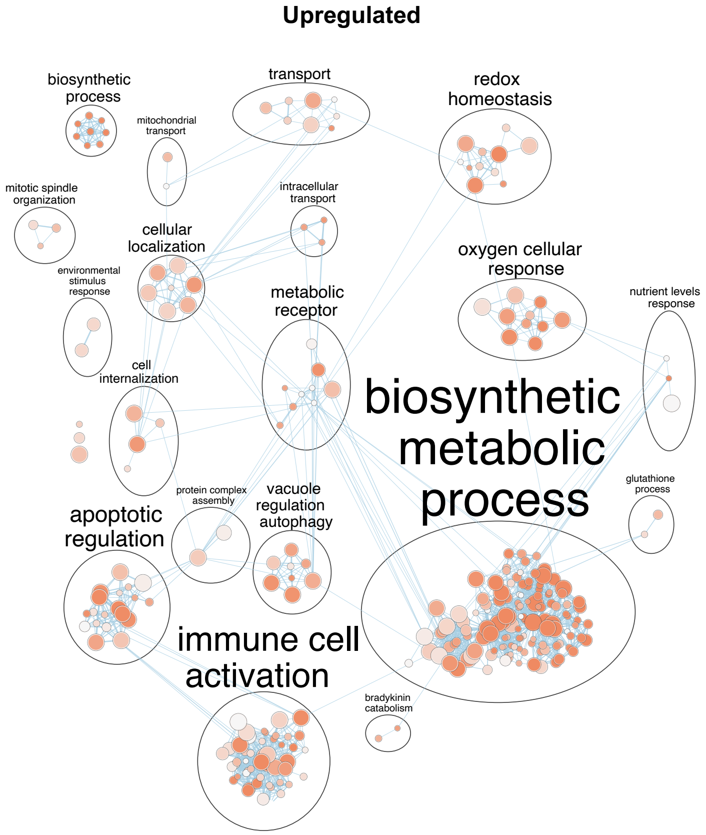
Upregulated
biosynthetic process
transport
redox homeostasis
mitochondrial transport
mitotic spindle organization
intracellular transport
cellular localization
environmental stimulus response
oxygen cellular response
metabolic receptor
nutrient levels response
cell internalization
biosynthetic metabolic process
protein complex assembly
vacuole regulation autophagy
apoptotic regulation
glutathione process
immune cell activation
bradykinin catabolism

B

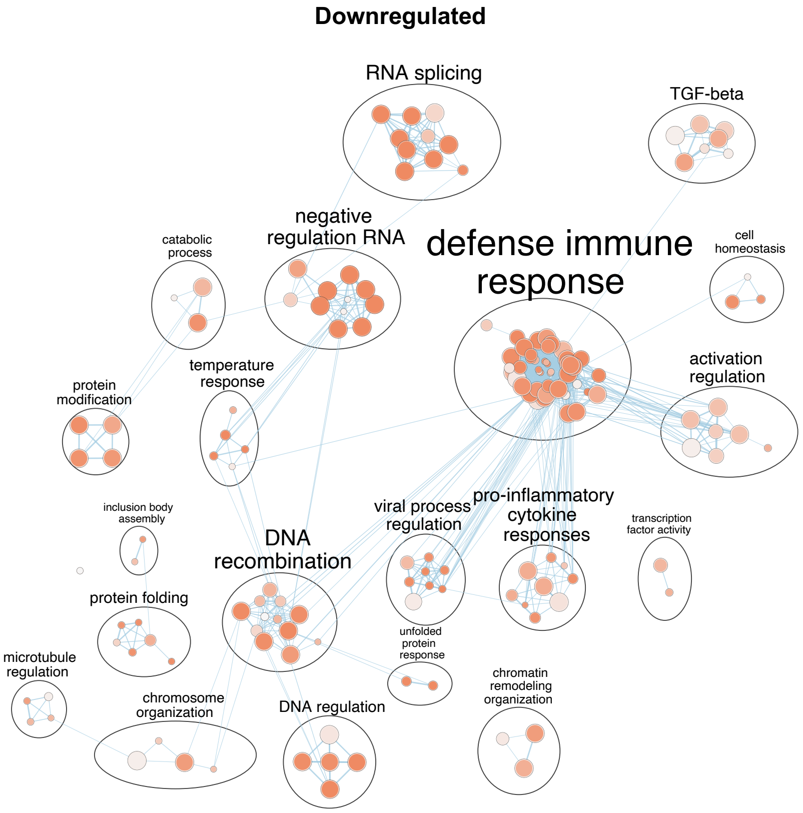
Downregulated
RNA splicing
TGF-beta
negative regulation RNA
catabolic process
defense immune response
cell homeostasis
temperature response
activation regulation
protein modification
inclusion body assembly
viral process regulation
pro-inflammatory cytokine responses
transcription factor activity
DNA recombination
protein folding
unfolded protein response
microtubule regulation
chromosome organization
DNA regulation
chromatin remodeling organization

Figure S7: Complete network of biological pathways within mTreg differentially expressed genes. Enrichment results were mapped as a network of gene sets in nodes for upregulated (A) or downregulated (B) genes in mTreg compared to control. The complete network for each case is depicted. Nodes are grouped and annotated by their similarity according to their related genes. The size of the node is proportional to the total number of genes within each gene set. Proportion of shared genes between pathways represented as the thickness of the edges between nodes. Red colour gradient of the node is related to the level of enrichment significance (FDR q-value < 0.05). Font is scaled by cluster size. Cytoscape (v3.10.3) and Enrichment Map (v3.5.0) were used for visualisation of the g:Profiler data on Gene Ontology terms (biological process).

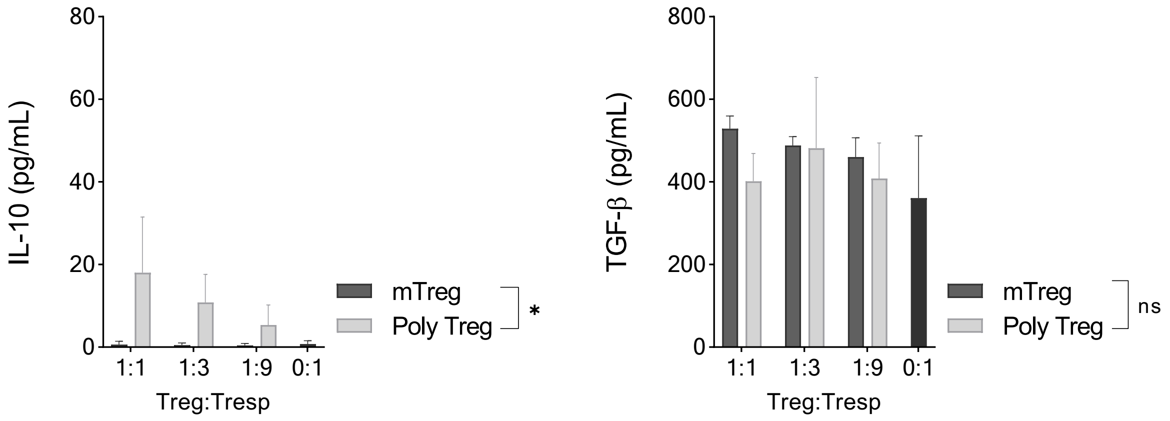

Figure S8: mTreg specific suppression does not rely on IL-10 and TGF-β secretion. SA were performed using Tcon as Tresp and oriDC as stimuli with Treg:Tresp comparing mTreg to polyclonal Treg from the same female sibling. IL-10 (left) or TGF-β (right) concentration in pg/mL was measured in the SA supernatant after 6 Days by multiplex analysis. Mean + SD of data obtained from three independent experiments are shown. Paired t-tests performed between mTreg vs Poly Treg groups. *, $p<0.05$; ns, statistically not significant.

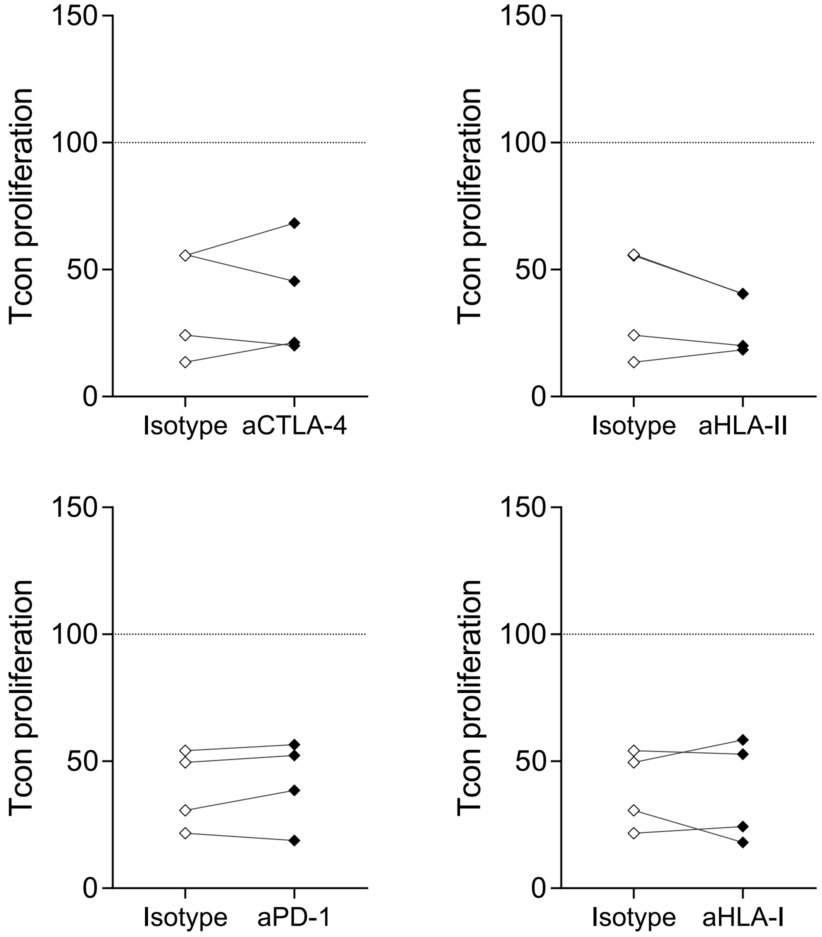

Figure S9: Suppression of CD4^{+} Tcon by mTreg *in vitro* may not depend exclusively on PD-1, CTLA-4, HLA class I or class II interactions. mTreg were pre-incubated with anti-CTLA-4, anti-PD-1, anti-HLA class I (aHLA-I) or anti-HLA class II (aHLA-II) blocking antibodies before being used in a regular SA of 1:3 Treg:Tcon ratio in the presence of oriDC as stimuli. Four independent experiments using different pairs of siblings are depicted. Paired t-tests were performed between isotype and blocking antibodies groups. All comparisons were statistically not significant.

Table S1: High-resolution HLA sequencing from fully HLA-matched siblings of opposite sexes and the third-party (3rd-pt) fully HLA-mismatched donors used to generate the data presented in each figure.

Figure	Donor	Sex	HLA-A	HLA-B	HLA-C	HLA-DRB1	HLA-DQA1	HLA- DQB1
Optimization	Treg	Female	A*02:01 A*68:02	B*07:05 B*40:01	C*03:04 C*07:02	DRB1*01:01 DRB1*13:04	DQA1*01:01 DQA1*05:05	DQB1*03:19 DQB1*05:01
	DC	Male	A*02:01 A*68:02	B*07:05 B*40:01	C*03:04 C*07:02	DRB1*01:01 DRB1*13:04	DQA1*01:01 DQA1*05:05	DQB1*03:19 DQB1*05:01
	3rd-pt DC	Male	A*01:01 A*23:01	B*51:01 B*58:01	C*07:01 C*15:02	DRB1*07:01 DRB1*07:01	DQA1*02:01 DQA1*02:01	DQB1*02:02 DQB1*02:02
Optimization	Treg	Female	A*01:01 A*30:01	B*35:08 B*49:01	C*04:01 C*07:01	DRB1*11:02 DRB1*11:73	DQA1*05:05 DQA1*05:05	DQB1*03:01/19 DQB1*03:19
	DC	Male	A*01:01 A*30:01	B*35:08 B*49:01	C*04:01 C*07:01	DRB1*11:02 DRB1*11:73	DQA1*05:05 DQA1*05:05	DQB1*03:01/19 DQB1*03:19
	3rd-pt DC	Male	A*24:02 A*68:02	B*40:01 B*78:01	C*03:04 C*16:01	DRB1*13:01 DRB1*13:01	DQA1*01:03 DQA1*01:03	DQB1*06:03 DQB1*06:03
2 and 4	Treg	Female	A*02:01 A*11:01	B*14:02 B*44:02	C*05:01 C*08:02	DRB1*01:02 DRB1*13:01	DQA1*01:01 DQA1*01:03	DQB1*05:01 DQB1*06:03
	DC	Male	A*02:01 A*11:01	B*14:02 B*44:02	C*05:01 C*08:02	DRB1*01:02 DRB1*13:01	DQA1*01:01 DQA1*01:03	DQB1*05:01 DQB1*06:03
	3rd-pt DC	Male	A*01:01 A*23:01	B*51:01 B*58:01	C*07:01 C*15:02	DRB1*07:01 DRB1*07:01	DQA1*02:01 DQA1*02:01	DQB1*02:02 DQB1*02:02
2	Treg	Female	A*26:01 A*33:01	B*08:01 B*15:03	C*02:02 C*07:02	DRB1*10:01 DRB1*10:01	DQA1*01:04 DQA1*01:04	DQB1*05:01 DQB1*05:01
	DC	Male	A*26:01 A*33:01	B*08:01 B*15:03	C*02:02 C*07:02	DRB1*10:01 DRB1*10:01	DQA1*01:04 DQA1*01:04	DQB1*05:01 DQB1*05:01
	3rd-pt DC	Male	A*01:01 A*23:01	B*51:01 B*58:01	C*07:01 C*15:02	DRB1*07:01 DRB1*07:01	DQA1*02:01 DQA1*02:01	DQB1*02:02 DQB1*02:02
2, 4	Treg	Female	A*11:01 A*11:01	B*44:02 B*58:01	C*05:01 C*07:01	DRB1*08:04 DRB1*12:01	DQA1*04:01 DQA1*05:05	DQB1*03:01 DQB1*04:02
	DC	Male	A*11:01 A*11:01	B*44:02 B*58:01	C*05:01 C*07:01	DRB1*08:04 DRB1*12:01	DQA1*04:01 DQA1*05:05	DQB1*03:01 DQB1*04:02
	3rd-pt DC	Male	A*24:02 A*32:01	B*15:01 B*35:03	C*03:03 C*04:01	DRB1*13:01 DRB1*13:02	DQA1*01:02 DQA1*01:03	DQB1*06:03 DQB1*06:04
2, 4, S3	Treg	Female	A*02:02 A*11:01	B*41:01 B*51:01	C*14:02 C*17:01	DRB1*13:01 DRB1*15:01	DQA1*01:02 DQA1*01:03	DQB1*06:02 DQB1*06:03
	DC	Male	A*02:02 A*11:01	B*41:01 B*51:01	C*14:02 C*17:01	DRB1*13:01 DRB1*15:01	DQA1*01:02 DQA1*01:03	DQB1*06:02 DQB1*06:03
	3rd-pt DC	Male	A*01:01 A*30:01	B*35:08 B*49:01	C*04:01 C*07:01	DRB1*11:02 DRB1*11:73	DQA1*05:05 DQA1*05:05	DQB1*03:01/19 DQB1*03:19
5	Treg donor	Female	A*02:01 A*24:02	B*44:02 B*51:01	C*05:01 C*14:02	DRB1*13:01 DRB1*14:01	DQA1*01:03 DQA1*01:04	DQB1*05:03 DQB1*06:03
	DC donor	Male	A*02:01 A*24:02	B*44:02 B*51:01	C*05:01 C*14:02	DRB1*13:01 DRB1*14:01	DQA1*01:03 DQA1*01:04	DQB1*05:03 DQB1*06:03
	3rd-pt DC donor	Male	A*01:01 A*23:01	B*49:01 B*50:01	C*06:02 C*07:01	DRB1*07:01 DRB1*08:03	DQA1*02:01 DQA1*06:01	DQB1*02:02 DQB1*03:01
2 to 5, S3 to S5	Treg	Female	A*02:01 A*24:02	B*44:02 B*51:01	C*05:01 C*14:02	DRB1*13:01 DRB1*14:01	DQA1*01:03 DQA1*01:04	DQB1*05:03 DQB1*06:03
	DC	Male	A*02:01 A*24:02	B*44:02 B*51:01	C*05:01 C*14:02	DRB1*13:01 DRB1*14:01	DQA1*01:03 DQA1*01:04	DQB1*05:03 DQB1*06:03
	3rd-pt DC	Male	A*33:01 A*66:01	B*14:02 B*41:02	C*08:02 C*17:03	DRB1*10:01 DRB1*13:03	DQA1*01:05 DQA1*05:05	DQB1*03:01 DQB1*05:01
3, 5, S3, S4	Treg	Female	A*32:01 A*33:01	B*49:01 B*78:01	C*07:01 C*16:01	DRB1*13:01 DRB1*15:01	DQA1*01:02 DQA1*01:03	DQB1*06:02 DQB1*06:03
	DC	Male	A*32:01 A*33:01	B*49:01 B*78:01	C*07:01 C*16:01	DRB1*13:01 DRB1*15:01	DQA1*01:02 DQA1*01:03	DQB1*06:02 DQB1*06:03
	3rd-pt DC	Male	A*02:01 A*02:01	B*18:01 B*41:02	C*07:02 C*17:03	DRB1*08:01 DRB1*13:03	DQA1*04:01 DQA1*05:05	DQB1*03:01 DQB1*04:02

3 to 5, S3 to S5	Treg	Female	A*24:02<br>A*24:02	B*15:03<br>B*45:01	C*16:01<br>C*16:01	DRB1*10:01<br>DRB1*13:01	DQA1*01:03<br>DQA1*01:05	DQB1*05:01<br>DQB1*06:03
	DC	Male	A*24:02<br>A*24:02	B*15:03<br>B*45:01	C*16:01<br>C*16:01	DRB1*10:01<br>DRB1*13:01	DQA1*01:03<br>DQA1*01:05	DQB1*05:01<br>DQB1*06:03
	3rd-pt DC	Male	A*02:01<br>A*68:02	B*14:02<br>B*15:03	C*02:02<br>C*08:02	DRB1*03:01<br>DRB1*15:01	DQA1*01:02<br>DQA1*05:01	DQB1*02:01<br>DQB1*06:02
4, 5, S3, S5	Treg	Female	A*03:01<br>A*03:01	B*18:01<br>B*55:01	C*01:02<br>C*03:03	DRB1*01:01<br>DRB1*10:01	DQA1*01:01<br>DQA1*01:05	DQB1*05:01<br>DQB1*05:01
	DC	Male	A*03:01<br>A*03:01	B*18:01<br>B*55:01	C*01:02<br>C*03:03	DRB1*01:01<br>DRB1*10:01	DQA1*01:01<br>DQA1*01:05	DQB1*05:01<br>DQB1*05:01
	3rd-pt DC	Male	A*02:01<br>A*24:02	B*38:01<br>B*51:01	C*12:03<br>C*15:02	DRB1*13:01<br>DRB1*13:01	DQA1*01:03<br>DQA1*01:03	DQB1*06:03<br>DQB1*06:03
3 to 5, S2 to S4	Treg	Female	A*23:01<br>A*33:01	B*08:01<br>B*14:02	C*07:02<br>C*08:02	DRB1*01:02<br>DRB1*13:02	DQA1*01:01<br>DQA1*01:02	DQB1*05:01<br>DQB1*05:01
	DC	Male	A*23:01<br>A*33:01	B*08:01<br>B*14:02	C*07:02<br>C*08:02	DRB1*01:02<br>DRB1*13:02	DQA1*01:01<br>DQA1*01:02	DQB1*05:01<br>DQB1*05:01
	3rd-pt DC	Male	A*29:02<br>A*30:02	B*18:01<br>B*44:03	C*05:01<br>C*16:01	DRB1*03:01<br>DRB1*03:01	DQA1*05:01<br>DQA1*05:01	DQB1*02:01<br>DQB1*02:01

Table S2: Differentially expressed genes in mTreg. – *attached file*

Table S3: Enrichment pathways within upregulated genes. – *attached file*

Table S4: Enrichment pathways within downregulated genes. – *attached file*

Table S5: List of primary antibodies.

Flow Cytometry				
**Target Ag**	**Clone**	**Conjugate**	**Supplier**	**Catalog #**
CD3	OKT3	PerCP-Cy5.5	Thermo Fisher Scientific (eBioscience)	45-0037-42
CD3	OKT3	FITC	Thermo Fisher Scientific (eBioscience)	11-0037-42
CD4	RPA-T4	BV785	BioLegend	300554
CD4	RPA-T4	APC	Thermo Fisher Scientific (eBioscience)	17-0049-42
CD8	RPA-T8	APC-e780	Thermo Fisher Scientific (eBioscience)	47-0088-42
CD25	2A3	PE	StemCell Technologies	60153PE
CD127	HIL-7R-M21	PE-Cy7	BD Biosciences	560822
CD154	24-31	BV711	BioLegend	310838
CD137	4B4-1	APC	BioLegend	309810
HLA-DR	LN3	APC-e780	Thermo Fisher Scientific (eBioscience)	47-9956-42
CD152	BNI3	PE-CF594	BD Biosciences	562742
CD279	EH12.1	BV605	BD Biosciences	563245
CD39	TU66	BV650	BD Biosciences	563681
Foxp3	PCH101	e450	Thermo Fisher Scientific (eBioscience)	48-4776-42
CD19	HIB19	FITC	Thermo Fisher Scientific (eBioscience)	11-0199-42
CD56	TULY56	FITC	Thermo Fisher Scientific (eBioscience)	11-0566-42
CD80	L307.4	PE	BD Biosciences	560925
CD83	HB15e	PE-Cy7	Thermo Fisher Scientific (eBioscience)	25-0839-42
CD86	IT2.2	PerCP-e710	Thermo Fisher Scientific (eBioscience)	46-0869-42
CD11c	B-ly6	APC	BD Biosciences	559877
CD14	61D3	e450	Thermo Fisher Scientific (eBioscience)	48-0149-42
CD274	29E.2A3	BV711	BioLegend	329722

Blocking antibodies				
**Target Ag**	**Clone**	**Conjugate**	**Supplier**	**Catalog #**
Purified NA/LE mouse anti-human PD-1 (CD279)	EH12.1	-	BD Biosciences	562138
Purified NA/LE mouse anti-human CTLA-4 (CD152)	BNI3	-	BD Biosciences	555850
Purified NA/LE mouse anti-human HLA-A/B/C	DX17	-	BD Biosciences	560187
Purified NA/LE mouse anti-human HLA-DR/DP/DQ	Tu39	-	BD Biosciences	555556
